# HMMR promotes prostate cancer proliferation and metastasis via AURKA/mTORC2/E2F1 positive feedback loop

**DOI:** 10.1038/s41420-023-01341-0

**Published:** 2023-02-07

**Authors:** Kaixuan Guo, Cheng Liu, Juanyi Shi, Cong Lai, Ze Gao, Jiawen Luo, Zhuohang Li, Zhuang Tang, Kuiqing Li, Kewei Xu

**Affiliations:** 1 Department of Urology, Sun Yat-sen Memorial Hospital, Sun Yat-sen University, Guangzhou, Guangdong P. R. China; 2 Guangdong Provincial Key Laboratory of Malignant Tumour Epigenetics and Gene Regulation, Sun Yat-sen Memorial Hospital, Sun Yat-sen University, Guangzhou, Guangdong P. R. China; 3 Guangdong Provincial Clinical Research Center for Urological Diseases, Guangzhou, Guangdong P. R. China; 4 Department of Hepatobiliary Surgery, Sun Yat-Sen Memorial Hospital, Sun Yat-Sen University, Guangzhou, Guangdong P. R. China

**Keywords:** Prostate cancer, TOR signalling

## Abstract

Although dysregulated HMMR is linked to prostate cancer (PCa) prognosis, the precise mechanisms remain unclear. Here, we sought to elucidate the role of HMMR in PCa progression as well as underlying mechanism. Herein, we found that upregulation of HMMR frequently observed in PCa samples and was associated with poor prognosis. Additionally, HMMR significantly promoted PCa proliferation and metastasis through gain- and loss-of function approaches in vitro and in vivo. Mechanistically, HMMR may interact with AURKA and elevated AURKA protein level through inhibiting ubiquitination-mediated degradation, which subsequently activated mTORC2/AKT pathway to ensure the reinforcement of PCa progression. Moreover, upregulated E2F1 caused from sustained activation of mTORC2/AKT pathway in turn function as transcription factor to promote HMMR transcription, thereby forming a positive feedback loop to trigger PCa progression. Importantly, administration of the mTOR inhibitor partially antagonised HMMR-mediated PCa progression in vivo. In summary, we not only reveal a novel possible post-translation mechanism mediated by HMMR involved in AURKA regulation, but also describe a positive feedback loop that contributes to PCa deterioration, suggesting HMMR may serve as a potential promising therapeutic target in PCa.

## Introduction

Prostate cancer (PCa) is one of the most prevalent tumours in males [1]. Although improvements in understanding of the biology have significantly prolonged the survival of PCa patients, this disease is the fifth leading cause of cancer-associated deaths in males [1–3]. Previous researches report that once localised PCa progress to an advanced stage, the survival rate decreased significantly [4, 5]. The media survival time of patients diagnosed with castration-resistant prostate cancer (CRPC) generally plummeted within 20 months [6, 7]. The sophisticated molecular mechanisms underlying PCa progression seriously restricted the improvement of strategy. Therefore, there is an urgent need to characterise novel therapeutic targets and mechanisms for PCa.

Hyaluronan-mediated motility receptor (HMMR), also termed CD168, was first described by Turley in murine cells [8]. It is reported that HMMR has an extensive coiled-coil structure (CC) that contains multiple sites for interactive partners [9, 10]. Initially, HMMR was considered a novel hyaluronan-mediated motility receptor and a microtubule-associated spindle assembly factor [8, 9, 11, 12]. Currently, abundant studies indicate that HMMR plays multiple functional roles in regulating proliferation and metastasis [13], maintenance of stemness [14] and chemotherapy resistance [15] in various tumours, such as lung cancer [16], liver cancer [17], bladder cancer [13] and gastric cancer [18]. Additionally, our previous study found that HMMR was elevated in PCa tumours tissues and associated with poor prognosis [19]. Although the oncogenicity of HMMR in PCa is preliminary disclosed, the precise role and mechanism remain largely poor understood, which deserves further explored.

mTOR is a crucial factor that participated in the various solid malignant tumours’ progression, and application of inhibitor of mTOR to delay tumour progression has become a promising strategy [20]. The diverse functions of mTOR stem from the phosphorylation of mTORC1 and mTORC2 on different substrates [21]. mTORC2 primarily responds to cell growth, cell survival and the actin cytoskeleton by promoting AKT phosphorylation [22]. It is worth noting that excessive activation of AKT was observed in 42% of localised PCa and in almost all metastatic tumours [23]. Therefore, studying mTOR/AKT pathway may contribute to improve prognosis of PCa patients.

Our previous study characterised HMMR as a crucial tumour driver in PCa [19]. Hence, we explored the precise role and mechanism of HMMR. In present study, we found that upregulated HMMR was closely associated with poor prognosis, advanced pathologic T stage, as well as higher gleason score. HMMR depletion or overexpression significantly blocked or accelerated cell proliferation, cell cycle transited, migration and invasion. Mechanistically, HMMR might interact with AURKA and elevated AURKA protein level by inhibiting ubiquitination, which subsequently resulted in the activation of the mTOR/AKT axis. Of note, the sustained activation of mTOR/AKT promoted the expression of E2F1, which in turn promoted the transcription of HMMR to form a positive feedback loop that collectively triggered PCa progression. Additionally, application of rapamycin in vivo antagonised the overgrowth elicited by HMMR overexpression.

## Results

### HMMR was overexpressed in PCa and associated with poor prognosis

**Fig. 1:**
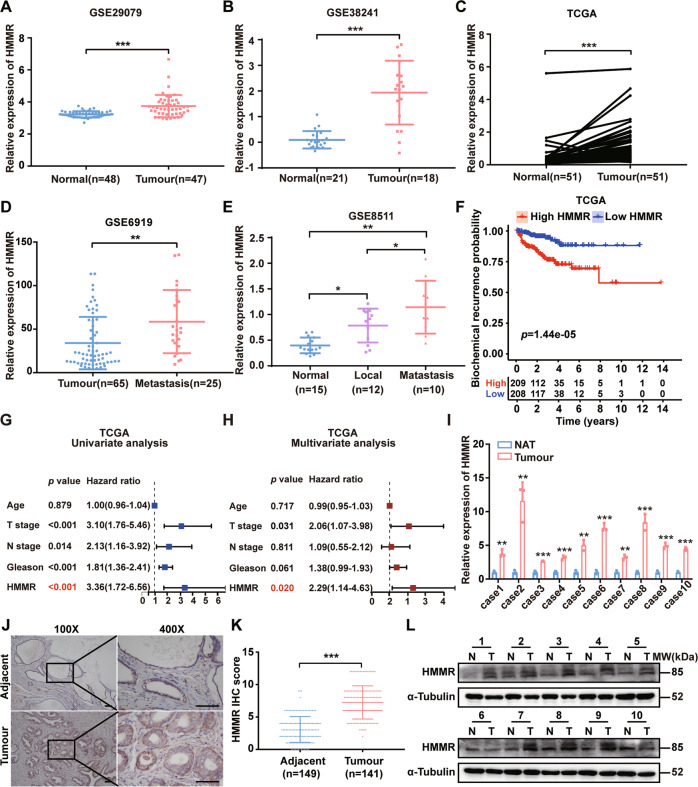
HMMR was overexpressed in PCa and associated with poor prognosis. **A**, **B** Analysis of HMMR mRNA expression in PCa and normal tissues (GSE29079, 38241). **C** Analysis of HMMR expression in 51 paired normal and PCa samples in TCGA**. D**, **E** HMMR mRNA expression in local PCa and metastatic tissues (GSE6919, 8511). **F** Biochemical recurrence risk assessment in TCGA stratified by HMMR expression. **G**, **H** Cox regression analysis (**G**: univariate; **H**: multivariate) to assess the correlation of HMMR expression and pathological indicators. **I** qRT–PCR analysis of HMMR mRNA in 10 paired PCa and adjacent tissues. **J**, **K** Representative IHC analysis of HMMR expression in PCa and adjacent tissues (**J**) and IHC quantification of HMMR expression in PCa and adjacent tissues (**K**), scale bar:100 μm. **L** Western blotting analysis of HMMR in 10 paired PCa and adjacent tissues. Data were presented as mean ± SD, *p < 0.05, **p < 0.01, ***p < 0.001.

Our previous research report that HMMR plays an important role in PCa [19]. Then the HMMR expression in PCa was analysed, and we found that HMMR upregulated in tumour tissues compared with adjacent tissues (Fig. 1A–C, E, Supplementary Fig. S1A). Importantly, overexpression of HMMR was also observed in metastatic tissues and lymph nodes, as well as positively correlated with Gleason score (Fig. 1D, E, Supplementary Fig. S1B, C). Concerning the prognosis of PCa, we found that upregulated HMMR had little significant value in judging overall survival (OS) but in judging the prognosis of patients with biochemical recurrence (BCR) (Fig. 1F, Supplementary Fig. S1D, E), univariate and multivariate analyses further indicated that HMMR was an independent risk factor in PCa (Fig. 1G, H). Additionally, analysis of clinical samples revealed that HMMR was overexpressed in tumour tissues (Fig. 1I–L). Moreover, after analysing in 124-case cohort, we found that high level of HMMR was positively associated with advanced T stage (*p* = 0.0198) and high gleason score (*p* = 0.0016) respectively (Table 1). Collectively, these findings preliminarily revealed that upregulated HMMR was closely associated with poor prognosis of PCa.

**Table 1:** Association between HMMR expression level and clinicopathological characteristics in 124 cases of PCa.

Clinical parameters	Patients number	HMMR		*P* value
		Low (*n* = 61)	High (*n* = 63)	
Age				
≤65	51	22	29	0.2595
>65	73	39	34	
T stage				
T1–2	60	36	24	0.0198*
T3–4	64	25	39	
N stage				
N0	114	59	55	0.0956
N1	10	2	8	
Gleason score				
<7	33	24	9	0.0016**
≥7	91	37	54	

Fisher’s exact test (N stage) and Chi-square test (Age, T stage, Gleason) were used for analysis, respectively. *p < 0.05, **p < 0.01.

### HMMR accelerated the progression of PCa

**Fig. 2:**
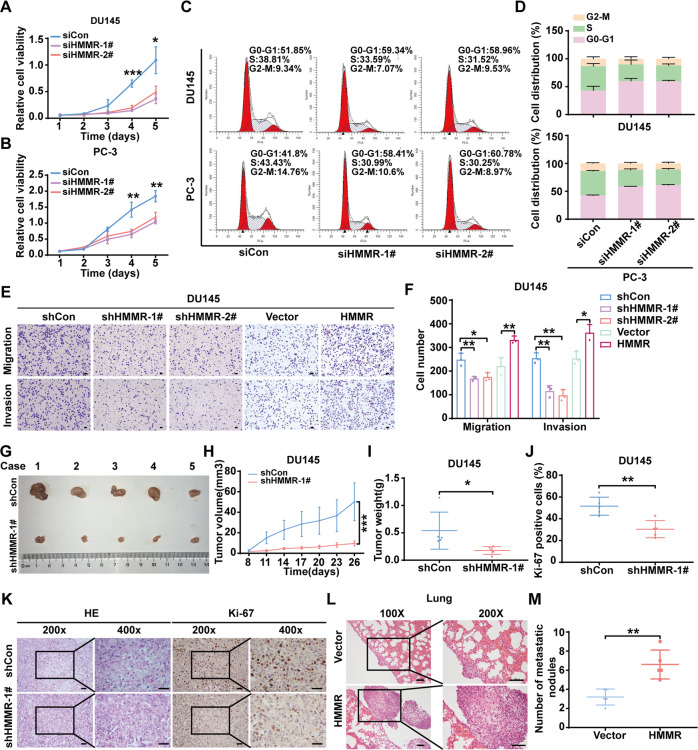
HMMR accelerated the progression of PCa in vitro and in vivo. **A**, **B** CCK-8 analysis of cell viability after transfecting DU145 (**A**) and PC-3 (**B**) cells with HMMR-specific siRNA (siHMMR-1#, siHMMR-2#) or negative control siRNA (siCon) at the indicated times. **C**, **D** Flow cytometry analysis of the ratio of HMMR-knockdown DU145 (upper panel) and PC-3 (lower panel) cells in the cell cycle phase. **E**, **F** Transwell analysis of cell migration and invasion after transfecting HMMR-specific siRNAs or overexpression plasmid, siCon and vector used as negative control respectively. **G** Gross observation of subcutaneous tumours. **H** Analysis of tumour volumes of the HMMR knockdown or control group recorded every three days. **I** Analysis of tumour weight of the HMMR knockdown or control group. **J**, **K** Quantitative analysis and representative IHC analysis of Ki-67-positive cells and HE staining derived from the shCon and shHMMR-1# groups, scale bar: 100 μm. **L**. HE staining of lung derived from metastasis model. **M** Analysis of metastatic nodules from the excised lungs of xenograft metastatic models. Data were presented as mean ± SD. *p < 0.05, **p < 0.01, ***p < 0.001.

To determine HMMR function in vitro, PCa cell lines with stable HMMR silencing were constructed, and the knockdown efficacy was validated (Supplementary Fig. S2A). CCK-8 assays showed that HMMR knockdown (KD) inhibited the proliferation of DU145 and PC-3 cells (Fig. 2A, B), whereas HMMR overexpression significantly facilitated the proliferation of PCa cells (Supplementary Fig. S2B, C). A similar trend also was observed in DU145, PC-3 and 22Rv1 cells (Supplementary Fig. S2D–F). Considering that previous studies have implied that HMMR is involved in the mitosis process [9], the cell cycle distribution was analysed. Flow cytometry showed that HMMR KD notably increased the proportion of G0-G1 phase cells while simultaneously decreasing the proportion of S phase cells (Fig. 2C, D). The G0-G1 phase arrest induced by HMMR silencing revealed that HMMR regulated the cell cycle and impaired cell proliferation. Acquiring the ability to metastasise is one of the features of malignant tumours. Hence, we investigated whether upregulated HMMR was attributed to cell migration and invasion. Transwell assays showed that HMMR KD inhibited cell migration and invasion in DU145, PC-3 and 22Rv1 cells, and the opposite phenomenon occurred when HMMR was overexpressed (Fig. 2E, F, Supplementary Fig. S2G–J). Wound scratch assay also suggested that HMMR KD weakened wound closure and that overexpressed HMMR expedited wound healing (Supplementary Fig. S2K, L).

To identify role of HMMR in vivo, PCa cells stably silenced with HMMR were constructed and injected into Balb/c nude mice to generate a subcutaneous model. As shown in Fig. 2G, tumours derived from shHMMR were significantly smaller than those derived from shCon. Additionally, HMMR depletion markedly suppressed tumour growth (Fig. 2H, I). Furthermore, analysis of IHC showed that the ratio of Ki-67-positive cells dramatically decreased in shHMMR cells compared with the negative control (Fig. 2J, K). Additionally, the role of HMMR in regulating PCa metastasis was also evaluated. As shown in Fig. 2L, M, overexpression of HMMR significantly promoted lung metastasis. In summary, HMMR boosted PCa progression in vitro and in vivo.

### HMMR promoted the activation of the mTORC2/AKT pathway

**Fig. 3:**
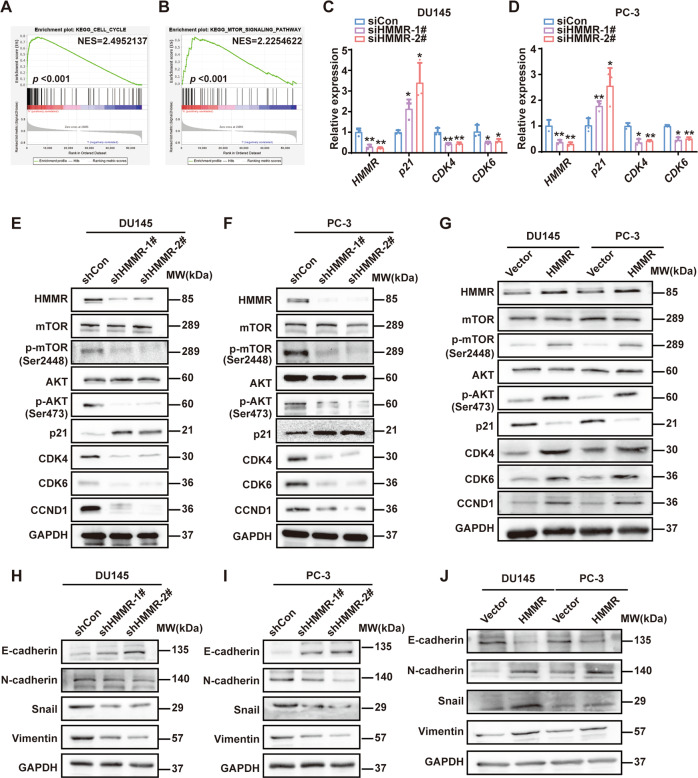
HMMR promoted the activation of the mTOR/AKT pathway. **A**, **B** GSEA analysis of the pathway in which HMMR might be involved (**A**: cell cycle; **B**: mTOR pathway). **C**, **D** qRT–PCR analysis of cell cycle-related proteins (P21, CDK4 and CDK6) in DU145 (**C**) and PC-3 (**D**) cells after transfection with siCon and siHMMR (#1, #2). **E**, **F** Western blotting analysis of crucial mTOR markers and downstream targets in DU145 (**E**) and PC-3 (**F**) cells after stable HMMR silencing (shHMMR-1#, shHMMR-2#). shCon was used as a negative control. **G** Western blotting analysis of mTOR pathway and downstream targets in DU145 and PC-3 cells after HMMR overexpression. **H**, **I** Analysis of EMT markers in DU145 (**H**) and PC-3 (**I**) cells with HMMR silencing. **J** Analysis of EMT markers in DU145 and PC-3 cells overexpressing HMMR. Data were presented as mean ± SD. *p < 0.05, **p < 0.01.

To explore the precise mechanism, gene set enrichment analysis (GSEA) based on TCGA was performed and we found that mTOR and cell cycle pathway were enriched significantly in HMMR-overexpressing cohorts (Fig. 3A, B). Hence, we hypothesise that HMMR might regulate the cell cycle through mTOR pathway. Accordingly, qRT–PCR and western blotting showed that p21 was upregulated, whereas CDK4, CDK6 and CCND1 were downregulated after HMMR silencing (Fig. 3C–F). Importantly, HMMR depletion led to phosphor-mTOR (Ser2448), phosphor-AKT (Ser473) decreased dramatically, and significant changes were not observed in the total mTOR or AKT (Fig. 3E, F). Then, HMMR overexpression plasmid was transfected into PCa cells for further verification, and the results showed that phosphor-mTOR and phosphor-AKT were upregulated. What is noteworthy is that our results further showed that it was mTORC2 that mediated the activation of HMMR/mTOR/AKT pathway (Supplementary Fig. S3A, B). In addition, decreased p21 and overexpressed CDK4/6 and CCND1 were also observed after HMMR overexpression (Fig. 3G). In summary, these data showed that HMMR regulated the cell cycle to facilitate the growth of PCa via mTORC2/AKT pathway. Previous studies indicated that epithelial-mesenchymal transition (EMT) regulated by mTORC2/AKT pathway may confer metastasis characteristics on primary tumours [24]. Thus, we subsequently studied the markers of EMT. As shown in Fig. 3H, I, HMMR knockdown resulted in a significant decrease in N-cadherin, snail and vimentin as well as an increase in E-cadherin. Additionally, the reverse phenomenon was observed after HMMR overexpression in PCa cells (Fig. 3J). Taken together, these results demonstrated that HMMR regulated the expression of cell cycle regulators or EMT markers via mTORC2/AKT pathway, thus mediating the progression of PCa.

### HMMR interacted with AURKA and mediated the mTORC2/AKT pathway

**Fig. 4:**
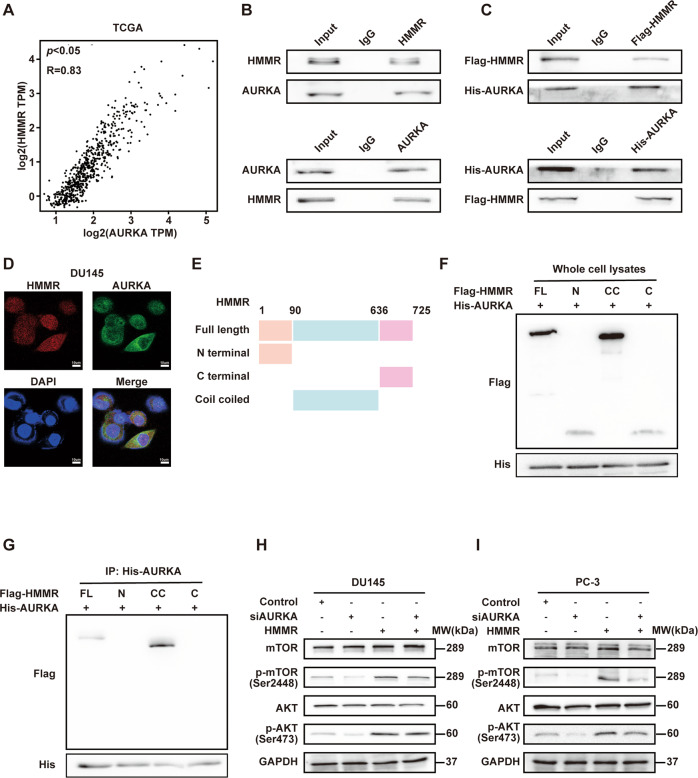
HMMR interacted with AURKA to mediate mTORC2/AKT pathway. **A** Correlation of HMMR and AURKA in PCa was analysed by GEPIA (*R* = 0.83, *p* < 0.05). **B** Co-IP analysis of the interaction of endogenous HMMR and AURKA with anti-HMMR (upper) or anti-AURKA (lower) antibodies. **C** Co-IP analysis of the interaction of Flag-labelled HMMR and His-labelled AURKA with anti-Flag (upper) or anti-His (lower), respectively. **D** Immunofluorescence was performed to analyse the relationship between HMMR and AURKA and their localisation in DU145 cells. The nucleus was stained with DAPI, scale bar: 10 μm. **E** Diagrammatic drawing of a series of HMMR deletion mutation plasmids. **F**, **G** Co-IP analysis of His-AURKA interacting with the detailed domain of Flag-HMMR. His-AURKA was co-transfected with HMMR deletion mutants in HEK-293T cells, and whole-cell lysates were immunoprecipitated with anti-His antibody and blotted with anti-Flag antibody. **H**, **I** Western blotting analysis of mTORC2/AKT pathway after transfection with AURKA-specific siRNA and/or HMMR plasmid in DU145 (**H**) and PC-3 (**I**) cells.

The mechanism of HMMR affects mTORC2/AKT axis was investigated subsequently. Through bioinformatics analysis, AURKA was predicted to be one of the crucial partners of HMMR (Supplementary Fig. S4A), and was strongly positively correlated with HMMR in PCa (Fig. 4A). Moreover, AURKA was upregulated in tumour tissues and associated with the BCR of PCa (Supplementary Fig. S4B, C). As presented in Fig. 4B, C, HMMR interacted with AURKA both exogenously and endogenously. Furthermore, immunofluorescence showed that HMMR and AURKA colocalized in the cytoplasm to varying degrees (Fig. 4D). To confirm the detailed binding region between HMMR and AURKA, we constructed a series of different deletion mutants of Flag-HMMR (Fig. 4E) and co-transfected them with His-AURKA in HEK-293T cells. Western blotting analysis showed that CC domain of HMMR interacted with AURKA (Fig. 4F, G). Additionally, emerging researches reports that AURKA functions as a kinase that phosphorylates multiple downstream targets to activate pathways, including mTORC2/AKT pathway [25, 26]. Therefore, we investigated whether the HMMR-induced mTORC2/AKT activation is AURKA dependent. After confirming the knockdown of AURKA (Supplementary Fig. S4D, E), we discovered that cell colony formation ability was remarkably inhibited and HMMR overexpression-induced overgrowth was also inhibited after AURKA depletion (Supplementary Fig. S5A, B), and a similar trend was observed in Transwell assay and scratch wound-healing assays (Supplementary Fig. S5C–F). We further studied the role of AURKA involved in mTOR/AKT axis. Phosphor-mTOR (Ser2448) and phosphor-AKT (Ser473) decreased after AURKA knockdown, and importantly, AURKA depletion partially controlled the overactive mTOR/AKT pathway (Fig. 4H, I). In conclusion, HMMR-induced overactivation of the mTORC2/AKT axis and corresponding phenotypic changes might be partially dependent on AURKA.

### HMMR evaluated AURKA protein level by inhibiting ubiquitination of AURKA

**Fig. 5:**
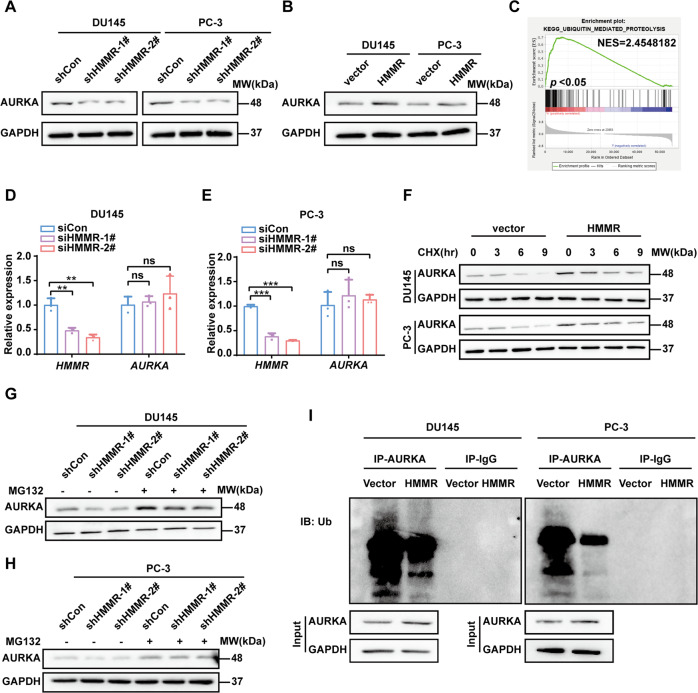
HMMR evaluated AURKA through inhibiting ubiquitination of AURKA. **A**, **B** Western blotting analysis of AURKA after HMMR knockdown (**A**) or overexpression (**B**). **C** GSEA analysis of the pathway in which HMMR might be involved. **D**, **E** qRT–PCR analysis of AURKA mRNA after transfecting siHMMR (#1, #2) or negative control in DU145 (**D**) and PC-3 (**E**) cells. **F** Western blotting analysis of AURKA levels after treating cells (vector and HMMR) with CHX at 0, 3, 6, and 9 h to assess the degradation ratio of AURKA. CHX concentration: 50 μg/ml, left panel: DU145, right panel: PC-3. GAPDH was used as a loading control. **G** Western blotting analysis of AURKA levels after treating DU145 (upper) and PC-3 (lower) cells with HMMR silencing or negative control cells with or without MG132; MG132 concentration: 100 nM. **H** Western blotting analysis of AURKA ubiquitination in HMMR-overexpressing or control cells. Whole-cell lysates were purified with anti-AURKA and then immunoprecipitated with anti-ubiquitin antibody, anti-IgG was used as a negative control. Data were presented as mean ± SD. **p < 0.01, ***p < 0.001, ns not significant.

We further investigated the actual role of HMMR/AURKA in PCa. As shown in Fig. 5A, HMMR silencing strikingly reduced the AURKA, and the opposite result was observed when HMMR upregulated (Fig. 5B). Whether HMMR affects AURKA protein level through transcriptional regulation or post-transcriptional regulation was concerned. However, our results demonstrated that HMMR rarely had a significant effect on AURKA transcription (Fig. 5D, E, Supplementary Fig. S4F). After reanalysis of GSEA, we found that HMMR involved in ubiquitination pathway (Fig. 5C). More importantly, HMMR has been identified as a substrate of APC/C complex [27]. Hence, we hypothesised that HMMR probably affect the stability of AURKA and enhance its expression through post-transcriptional modification. We treated HMMR overexpressed cells with CHX to block de novo protein synthesis. As shown in Fig. 5F, HMMR overexpression enhanced AURKA at the initial time, and the degradation rate notably slowed when HMMR overexpressed, which suggested that HMMR might have little effect on protein translation but improved the stability of AURKA. Then, MG132 was used to curb the ubiquitination to explore whether HMMR impaired AURKA ubiquitination. We found that treating cells with MG132 stably silencing HMMR conspicuously impeded AURKA degradation (Fig. 5G, H). These results indicated that HMMR might increase AURKA level by inhibiting degradation. More importantly, HMMR overexpression decreased the ubiquitination levels of endogenous AURKA in PCa cells (Fig. 5I). Taken together, HMMR may enhance the AURKA protein level by inhibiting ubiquitination.

### HMMR upregulated E2F1 and in turn promoted HMMR transcription to form a positive feedback loop

**Fig. 6:**
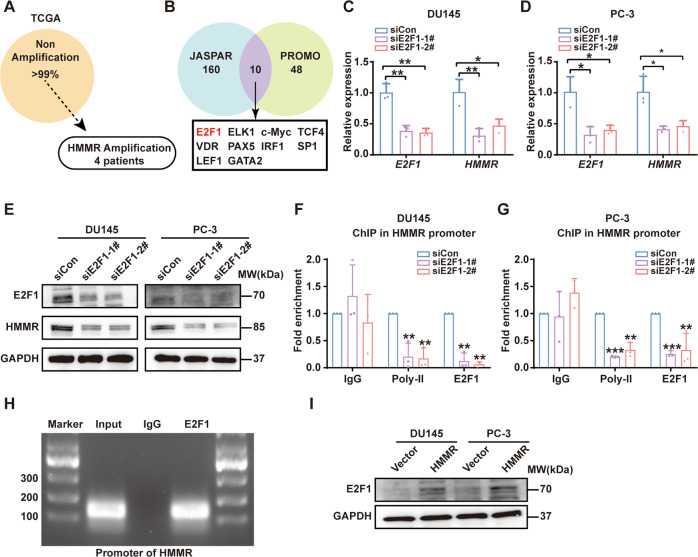
HMMR upregulated E2F1 and in turn promoted HMMR transcription to form a positive feedback loop. **A** Analysis of HMMR amplification mutation in TCGA. **B** Potential upstream TFs of HMMR predicted by JASPAR and PROMO. **C**, **D** qRT–PCR analysis of HMMR mRNA after transfection with E2F1-specific siRNA (siE2F1-1#, siE2F1-2#) in DU145 (**C**) and PC-3 (**D**) cells. **E** Western blotting analysis of HMMR after E2F1 knockdown. **F**–**H** ChIP-qPCR (**F**–**G**) and gel electrophoresis (**H**) analysis of Pol-II status in the promoters of HMMR and HMMR transcriptional activity after E2F1 knockdown in DU145 cells. **I** Western blotting analysis of E2F1 in the HMMR overexpression group of DU145 and PC-3 cells. *p < 0.05, **p < 0.01, ***p < 0.001.

Although the tumorigenic mechanism of HMMR was elucidated, upstream regulatory factors of HMMR remain unclear. In present study, we found that HMMR overexpression was seemingly not caused by amplification mutation (Fig. 6A). Therefore, we analysed the HMMR promotor region and predicted potential TFs in JASPAR and PROMO. A total of ten TFs (E2F1, ELK1, c-Myc, TCF4, VDR, PAX5, IRF1, SP1, LEF1 and GATA2) were screened (Fig. 6B). Then, the relationship between HMMR and TFs and the expression pattern of TFs in PCa were analysed. The results suggested that E2F1 had the strongest positive correlation with HMMR (Supplementary Fig. S6A-J), and E2F1 was frequently upregulated in PCa tissues and predicted poor prognosis (Supplementary Fig. S6K, L). To further validate the E2F1/HMMR regulatory network, small interfering RNAs targeting E2F1 were transfected and qRT–PCR showed that HMMR mRNA expression was remarkably decreased in DU145 and PC-3 cells when E2F1 was knocked down (Fig. 6C, D), and a similar trend was observed in HMMR protein levels (Fig. 6E). ChIP assay was applied for further verification immediately. As shown in Fig. 6F–H, the enrichment of the promotor region of HMMR was significantly reduced when E2F1 knockdown. These data indicated that E2F1 promoted HMMR transcription by binding to the promoter region. Intriguingly, it is reported that E2F1 is regulated by the mTOR/AKT pathway [28]. Consequently, we detected E2F1 levels by disturbing HMMR in PCa, and the results revealed that overexpressed HMMR promoted E2F1, which suggested that E2F1 was also a target of HMMR (Fig. 6I).

### mTOR inhibitor partly abolished the oncogenic role of HMMR

**Fig. 7:**
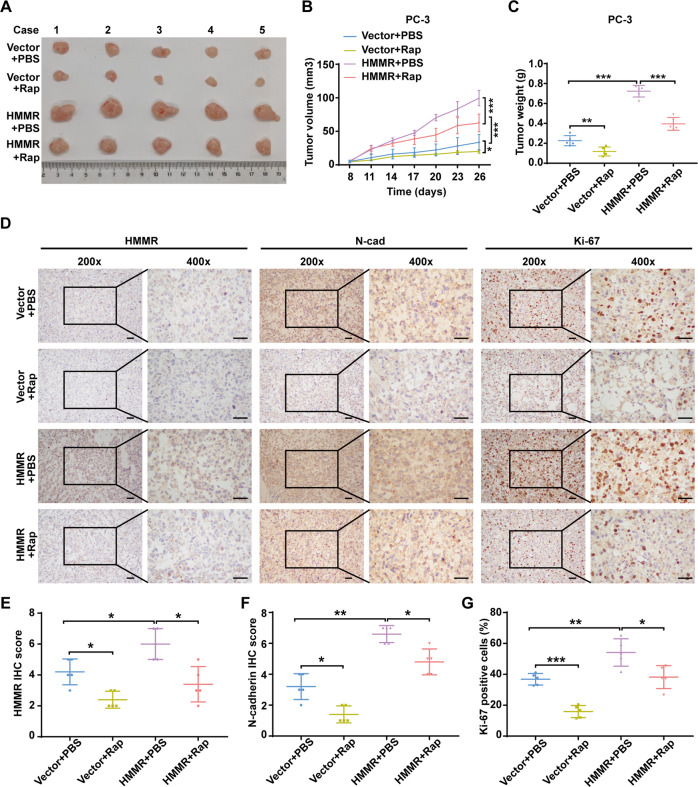
mTOR inhibitor partly abolished the oncogenic role of HMMR. **A** Gross observation of subcutaneous tumours in the vector and HMMR overexpression groups treated with/without rapamycin. **B** Analysis of tumour volumes of the indicated subcutaneous tumours recorded every three days. **C** Analysis of tumour weight of the indicated subcutaneous tumours. **D** Representative images of HMMR, N-cadherin, and Ki-67 of the indicated subcutaneous tumours; scale bar: 100 μm. **E**–**G** Quantification of HMMR (**E**), N-cadherin (**F**) expression, and proliferation index (**G**) in the indicated xenograft tumours, scale bar:100 μm. Data were presented as mean ± SD. *p < 0.05, **p < 0.01, ***p < 0.001.

Several mTOR inhibitors have been widely applied for tumour treatment [29–31]. We subsequently explored whether rapamycin exerted anticancer efficacy in vivo. PC-3 cells with HMMR stably overexpressing were used to construct the subcutaneous xenograft model [23], and the inhibitor of mTOCR2/AKT axis (Rapamycin:10 mg/kg) was used to treat the tumour-bearing mice [32]. Upregulated HMMR notably accelerated tumour growth, and rapamycin alone inhibited tumour growth. Importantly, by analysing tumour size and volume, we found that rapamycin significantly inhibited the proliferation of HMMR overexpressed tumour-bearing mice (Fig. 7A–C). In addition, analysis of IHC of isolated subcutaneous tumours clearly showed that rapamycin partly reduced the increased growth index caused by HMMR (Fig. 7D, G). In addition, we found that N-cadherin was upregulated by HMMR overexpression, which was consistent with our previous results, while it markedly fell off following treatment with rapamycin (Fig. 7D, F). Notably, the expression trend of HMMR aroused our interest. We found that HMMR expression was significantly downregulated when rapamycin was administered (Fig. 7D, E), which indicated that HMMR was in return regulated by the mTORC2/AKT pathway.

In summary, these findings suggested that a positive feedback loop of HMMR/E2F1 mediated by the AURKA/mTORC2/AKT axis may trigger PCa progression (Supplementary Fig. S7).

## Discussion

Frequent overexpression of HMMR has been observed in various solid tumours and is linked with malignant behaviours and poor prognosis, including bladder cancer, pancreatic cancer, liver cancer, lung cancer and PCa [13, 16–18, 33]. Nevertheless, the role of HMMR and mechanism underlying PCa progression remains elusive. In present study, we discovered that HMMR remarkably upregulated in PCa tissues and was positively associated with T stage and gleason score. Functionally, HMMR facilitated cell growth, migration and invasion in androgen receptor (AR) positive and negative cells, which in accord with previous results [16, 34]. More importantly, we revealed that HMMR might interacted with AURKA and improved AURKA protein level, which activated mTORC2/AKT pathway to mediate PCa progression. Consequently, the upregulation of E2F1 mediated by mTORC2/AKT in turn promoted HMMR transcription and consequently formed a positive feedback loop that triggered PCa progression. These results suggested that HMMR might be a crucial oncogenic regulator and a promising treatment target for PCa. However, the precise role and mechanism of HMMR in AR (+) cells and AR signalling deserved further investigating.

Disorders of cyclin-dependent kinases (CDKs) and cyclin-dependent kinase inhibitors (CKIs) are responsible for the difference in cell growth [35]. Hence, targeting cell cycle checkpoint has been a promising strategy to suppress tumour overgrowth [36]. Several inhibitors have been successfully developed for treating breast carcinoma [37], non-small-cell lung carcinoma [38] and prostate carcinoma [39]. In present study, we found that HMMR accelerated cell cycle transition and promoted tumour growth via regulating CDK4/6 and p21. These results not only prove that HMMR may be a crucial upstream regulator of CDKs or CKIs but also reveal a novel mechanism by which HMMR may be involved in prostate carcinogenesis. Although this regulatory relationship was preliminary verified in vitro, it is unknown whether the combined application of CDK inhibitors with HMMR inhibitors could achieve a more satisfactory effect, which deserves further investigation. In addition to proliferation, metastasis is considered the terminal step of PCa progression and is responsible for the majority of tumour-associated deaths [40]. EMT is a considerable characteristic of distant metastasis of tumours [20]. In the present study, we found that HMMR silencing prominently impaired the migration and invasion of PCa cells and restrained the expression of N-cadherin, vimentin and snail, which indicated that HMMR might be an important factor that regulated the PCa metastasis.

Aberrate activation of mTORC2/AKT has been a high-frequency adverse event in PCa [41]. mTORC2/AKT participates in multiple biological processes, such as proliferation and chemotherapy resistance, DNA damage repair and cell survival, metabolism and metastasis [42]. Consequently, developing potent mTORC2/AKT inhibitors is an ideal strategy. Consistent with previous researches, our results proved that rapamycin significantly limited PCa growth and partially eliminated the overgrowth caused by HMMR. However, due to the limitations of animal models, the experimental results are worth further verification in genetically engineered mouse models. At the same time, the development and use of small molecule inhibitors of HMMR will make this conclusion more convincing. Although animal results in this study are encouraging, few mTOR-specific inhibitors have achieved satisfactory effects in clinic. For instance, George DJ et al. found that everolimus alone had no clinical utility in men with mCRPC;[29] Deaglan J McHugh et al. demonstrated that temsirolimus plus cixutumumab displayed moderate anticancer effect but a non-negligible toxicity in mCRPC [43]; and Dana E Rathkopf proved that the combination of gefitinib and everolimus didn’t provide satisfactory benefit to mCRPC patients [44]. The clinical failure of mTOR inhibitors alone or in combination has created a great contradiction with scientific theory. In this research, we clarified that overexpressed HMMR remarkably promoted mTOR phosphorylation at Ser2448 and AKT phosphorylation at Ser473 in a partially AURKA-dependent manner, which shed light on another pathway of mTORC2/AKT activation and further strengthened the theoretical basis for the application of mTOR inhibitors to control tumour progression. Nevertheless, to resolve the contradiction between theory and clinical practice, further research is urgently needed.

AURKA is a crucial member of the serine/threonine kinase family that possesses diverse biological functions by phosphorylating different substrates [45]. Specifically, increasing studies have found that AURKA can regulate Wnt/β-catenin, NF-κB, Hippo and mTOR/AKT pathways [26, 46–48]. The involvement of all of these crucial cancer-related pathways revealed the fundamental role of AURKA in tumorigenesis and progression. Yong-Won Kwon et al reported FBXW7 as an E3 ligase for AURKA ubiquitination and degradation [49]. In present study, our results implied that HMMR likely increased AURKA expression by inhibiting ubiquitination, which described a novel post-translation modification for HMMR/AURKA. Despite these findings, the details involved in AURKA degradation are largely poorly understood. Therefore, the precise mechanisms of HMMR in AURKA ubiquitination deserve further investigation.

Previous research successfully identified FoxM1 as an upstream TF of HMMR in bladder cancer [13]. In the present study, after confirming the low frequency of genetic mutations of HMMR in PCa, we hypothesised that overexpression of HMMR in PCa perhaps resulted from TFs regulation. The promoter region of HMMR was analysed and potential TFs were subsequently predicted. E2F1 was screened and ChIP analysis further confirmed that E2F1 significantly promoted HMMR transcription by interacting with the promoter region of HMMR. Interestingly, previous researches report that E2F1 is a momentous target of AKT [28, 50], and we also confirmed that hyperactivation of mTORC2/AKT arising from HMMR overexpression increased E2F1 protein level. Our results not only clarified a novel mechanism of HMMR regulation, but also revealed a positive feedback loop composed with HMMR and E2F1, which provided more support for other studies [33], thusly providing a deeper understanding of HMMR.

## Materials and methods

### Patients and clinical samples

PCa tumour samples and matched adjacent tissues were collected from PCa patients who had undergone radical prostatectomy between January 2010 and January 2020 in the Department of Urology, Sun Yat-sen Memorial Hospital, Sun Yat-sen University (Guangzhou, China). All the samples were confirmed by pathological diagnosis and frozen in RNAlater at −80 °C once acquired. This study was approved by the Ethical Review Committee of Sun Yat-sen Memorial Hospital, and all patients had signed informed consent.

### Cell lines, cell culture, and drug treatment

Human PCa cell lines (DU145, PC-3) and HEK-293T cells were purchased from the American Type Culture Collection (ATCC, Manassas, VA, USA) and cultured in recommended medium supplemented with 10% foetal bovine serum (Invitrogen, Carlsbad, USA) according to recommendations by the ATCC. For the drug treatment, cycloheximide (CHX) and MG132 purchased from Selleck (Houston, TX, USA) were used in this study. The concentrations used were as follows: 50 μg/mL CHX and 100 nM MG132. All cells were cultured at 37 °C and 5% CO2, and violent shaking was avoided during culture.

### RNAi transfection, mRNA extraction, and qRT–PCR

Small interfering RNAs (siRNAs) targeting HMMR, AURKA and E2F1 were designed and synthesised by RiboBio (Guangzhou, China), and a detailed siRNA sequence is listed in Supplementary Table 1. Information on the primers is listed in Supplementary Table 2. For details, the procedures are presented in Supplementary file 1.

### Plasmid construction, extraction and transfection and Dual-luciferase reports assay

The HMMR overexpression plasmid, AURKA overexpression plasmid, corresponding serious depletion mutation plasmid, and empty vector was constructed by IGE BIO (Guangzhou, China). The plasmid was amplified by E. coli and extracted under the instructions of a standard protocol provided by the EndoFree Plasmid Midi Kit (CWBIO, China). For plasmid transfection, Opti-MEM™ medium (Thermo Fisher, USA) and X-tremeGENE™ HP DNA Transfection Reagent (Roche, Switzerland) were applied, and the transfection process was performed as suggested. For the Dual-luciferase reports assay, the procedures are presented in Supplementary file 1.

### Cell viability measurement, cell cycle analysis, wound healing and transwell assay

CCK-8 and colony formation assays were performed to detect cell viability. Propidium iodide (PI) staining was performed for a cell cycle distribution analysis. Transwell assays and wound healing assays were carried out to analyse cell migration and invasion ability. Detailed procedures are presented in Supplementary file 1.

### Western blotting, coimmunoprecipitation (Co-IP) and antibody reagents

Collected cells were lysed with an appropriate volume of RIPA on ice for 30 min, and the protein concentration was measured by Protein Assay Kit (Thermo Fisher, USA) after centrifugation. Protein was separated with 7.5–12% SDS-PAGE gel electrophoresis and constantly transferred onto PDVF membranes (0.22 μm, Millipore, Billerica, MA, USA). The membranes were successively incubated with the indicated primary antibodies and secondary IgG, then visualised with a chemiluminescence imaging system. The primary antibody reagents were listed in Supplementary file 1.

### Immunofluorescence (IF)

Cells were counted (5 × 10^4^) and seeded into confocal dishes. Cells were fixed with paraformaldehyde (Servicebio, Wuhan, China) after washing with iced phosphate-buffered saline (PBS) three times. The cells were incubated with 0.1% Triton for 10 min at room temperature (RM) and washed with iced PBS three times. After blocking with 1% BSA (1 h at RM), the cells were sequentially incubated with indicated antibody (overnight at 4 °C) and fluorescently labelled secondary antibody (1 h at RM avoiding light). Finally, the cells were incubated with 0.1 mg/mL DAPI for nuclear staining (10 min at RM avoiding light). The images were acquired and analysed with a ZEISS LSM800 confocal microscope (Carl Zeiss AG, Oberkochen, Germany). Antibodies and reagents used for IF were as follows: anti-HMMR (87129 S, 1:100), anti-AURKA (12100 S, 1:100, CST), DAPI (C1002, 1:10 000, Beyotime Biotechnology Ltd., Shanghai, China), Alexa Fluor® 647 (ab150115, 1:1000, Abcam, Cambridge, UK) and Alexa Fluor® 488 (ab150077, 1:1000, Abcam, Cambridge, UK).

### Animal experiments

All animal experiments were approved by the Animal Ethics Committee of Sun Yat-sen University and conducted in the Animal Experiment Centre of Sun Yat-sen University (Sun Yat-sen University, Guangzhou, China). Balb/c nude male mice were randomly divided into two or four groups (five mice peer group). Double-blind was done. PCa cells (3 × 10^6^/100 µl) with stable HMMR silencing or the negative control were prepared with 1× PBS and mixed with Matrigel (BD Biosciences) in equal volumes, and then the cell mixture was subcutaneously injected into Balb/c nude mice. The growth state of the mice, as well as the tumour volumes, were recorded every 3 days. After 26 days of observation, all the mice were sacrificed for isolating tumour specimens for analysis.

For rapamycin treatment, PCa cells with stable HMMR overexpression or empty vector were constructed and used as described above. After 7 days of injection, sterile 1× PBS or rapamycin (10 mg/kg) was injected into the mice every 3 days. After 26 days of observation, all the mice were sacrificed, and the volume and weight of specimens were recorded and analysed.

### Haematoxylin-eosin (HE) staining and immunohistochemistry (IHC)

All steps were conducted as described in the previous study [23]. All tissue specimens were fixed with 37% formalin and then paraffin-embedded, haematoxylin and eosin were used for staining. For IHC, the antibodies were listed as follows: HMMR (1:300, Proteintech, Wuhan, China), phosphor-AKT (Ser473) (1:100, Service Bio, Wuhan, China), N-cadherin (1:100, Service Bio, Wuhan, China), and Ki-67 (1:100, Service Bio, Wuhan, China). All images were captured by an ECLIPSE Ti microscope system (Nikon, Japan).

### Chromatin immunoprecipitation (ChIP)

Cells (1 × 10^7^) were harvested and crosslinked with 1% formaldehyde and 1% glycine solution according to the protocol provided by the Pierce magnetic ChIP kit (Thermo Scientific, USA). The cells were lysed with membrane extraction buffer, digested with MNase and sonicated for DNA fragmentation. The size of the fragments was verified by agarose electrophoresis and then respectively rotating incubated with anti-IgG, anti-E2F1, and anti-RNA polymerase-II antibodies overnight at 4 °C. The antibody-DNA fragment complex was purified with protein A/G magnetic beads and elution buffer. The enrichment of specific DNA fragments was analysed by qRT–PCR. Anti-GAPDH and RNA polymerase-II antibodies were provided by a Pierce magnetic ChIP kit, and anti-E2F1 (3742 S) was purchased from Cell Signalling Technology (MA, USA). Primers for amplifying HMMR promoter region are listed in Supplementary Table 2.

### Statistics

All statistical analysis were calculated by GraphPad Prism 7.0 (GraphPad Software, Inc, San Diego, CA, USA). All experiments were carried out three times, and the data are presented as the means ± SD. For normally distributed data, two-tailed Student’s *t* test and one-way or two-way analyses of variance were adopted. For nonnormally distributed data, a non-parametric Mann–Whitney test was applied. Survival analysis and Cox regression analysis were analysed with the Kaplan–Meier method and evaluated by the log-rank test performed by R (3.6.3 version, www.r-project.org). *p* < 0.05 was considered statistically significant.

## Supplementary information

Original western blotting

Supplementary materials and methods

Supplementary table 1

Supplementary table 2

Supplementary Fig. S1

Supplementary Fig. S2

Supplementary Fig. S3

Supplementary Fig. S4

Supplementary Fig. S5

Supplementary Fig. S6

Supplementary Fig. S7

Supplementary figure legends

## Data Availability

Publicly datasets analysed in this study were downloaded from TCGA (https://portal.gdc.cancer.gov/) and GEO (GEO: https://www.ncbi.nlm.nih.gov/geo/).
