# Supplementary material for: HMMR promotes prostate cancer proliferation and metastasis via AURKA/mTORC2/E2F1 positive feedback loop
Source: Cell Death Discov. 2023 Feb 7;9:48. doi: 10.1038/s41420-023-01341-0 (PMC9905489; doi:10.1038/s41420-023-01341-0)

Fig. 1L

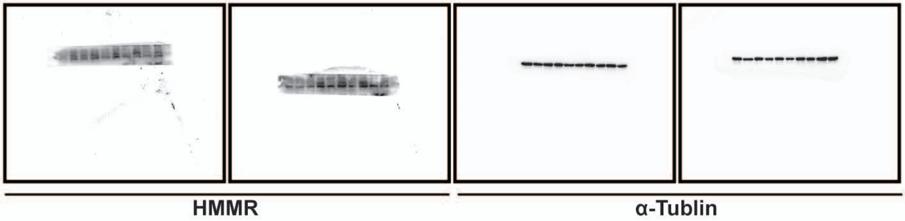

Fig. 3E

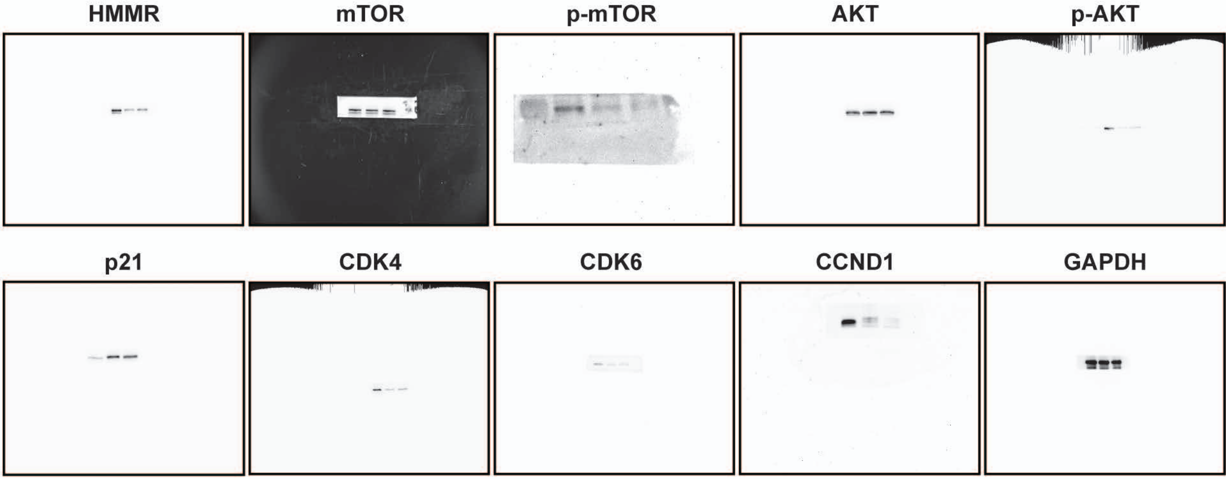

Fig. 3F

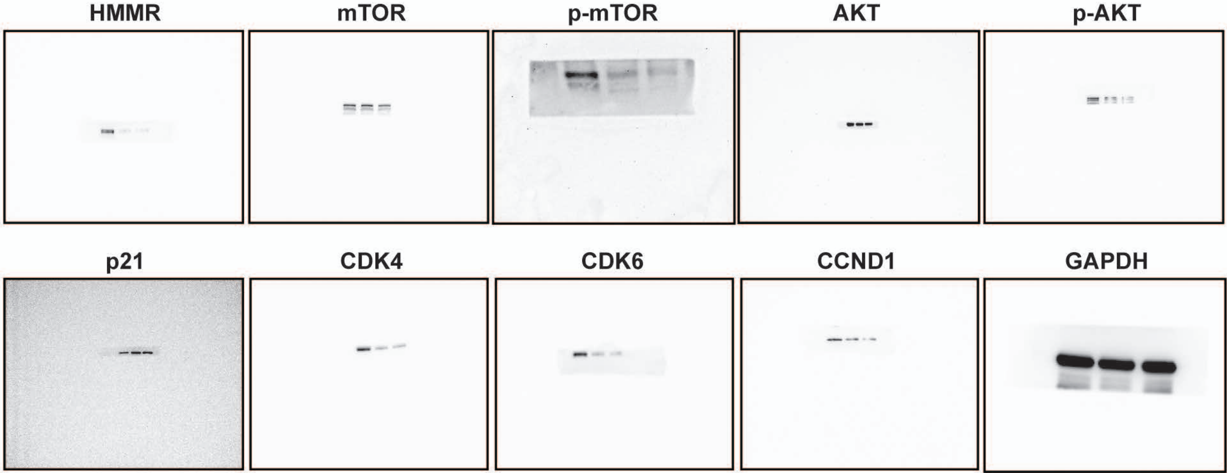

Fig. 3G

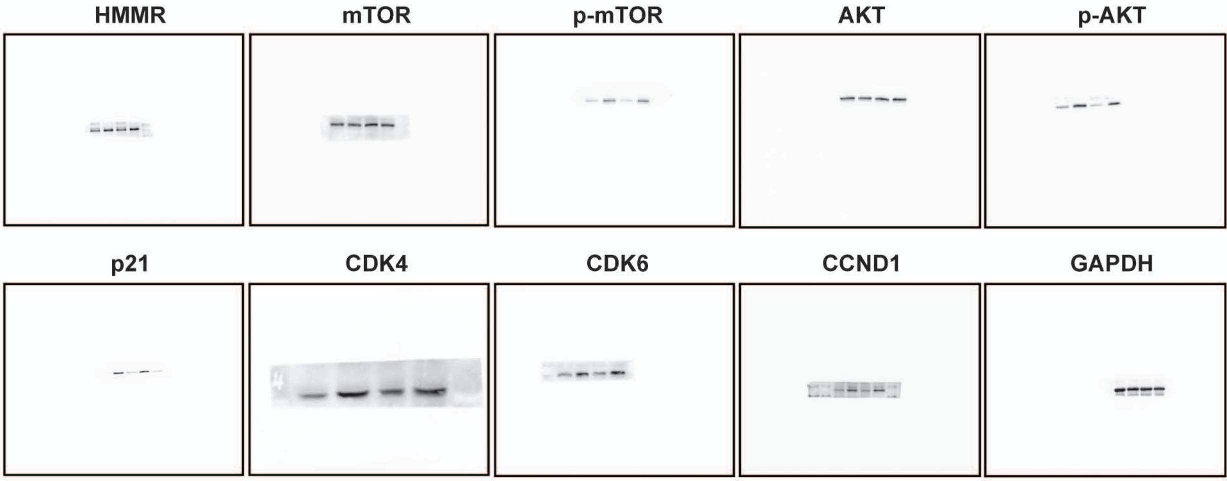

Fig. 3H

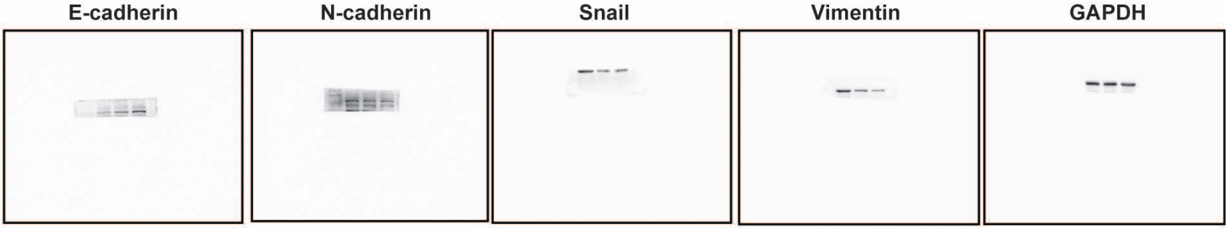

Fig. 3I

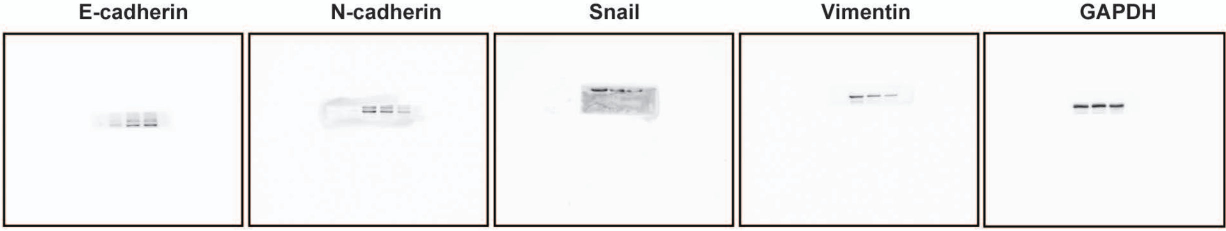

Fig. 3J

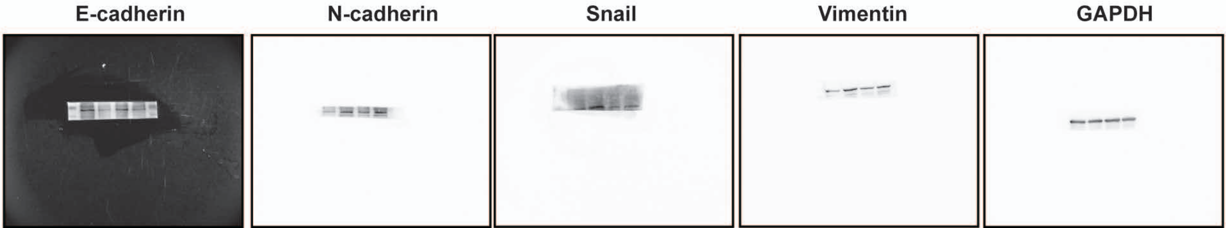

Fig. 4B

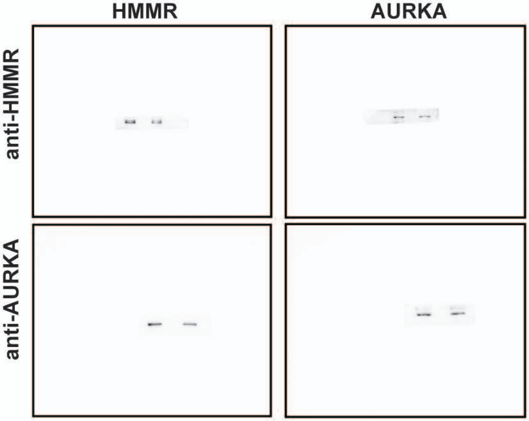

Fig. 4C

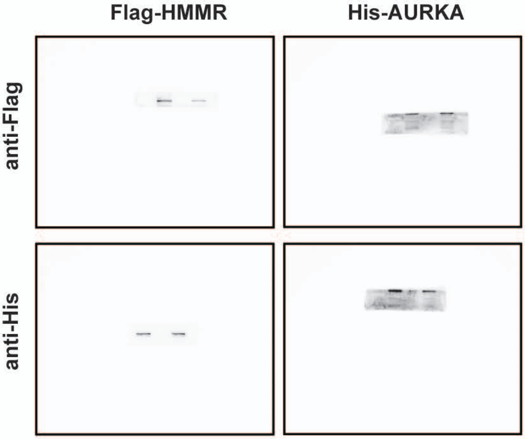

Fig. 4F

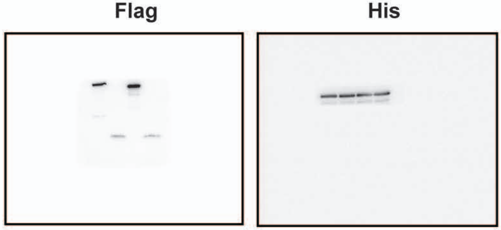

Fig. 4G

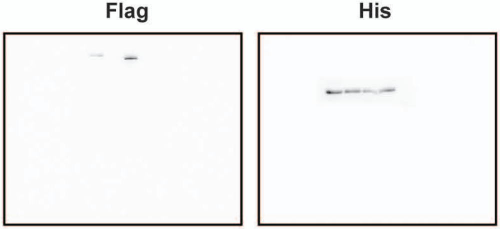

Fig. 4H

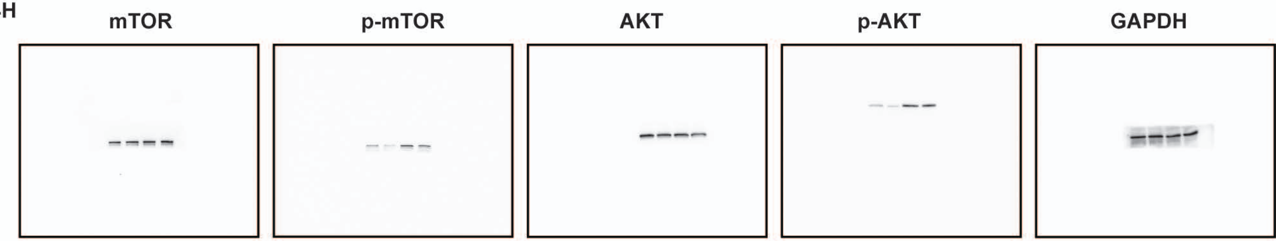

Fig. 4I

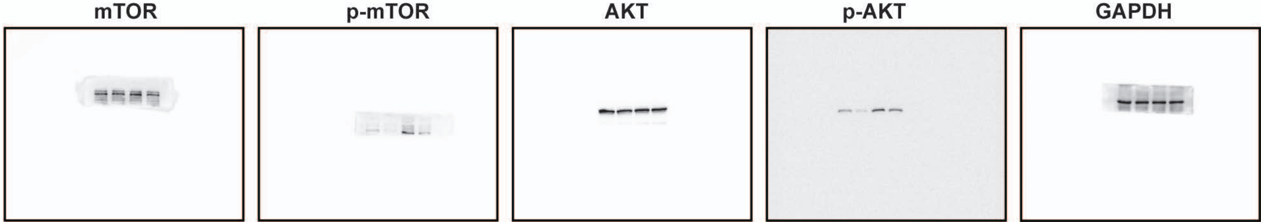

Fig. 5A

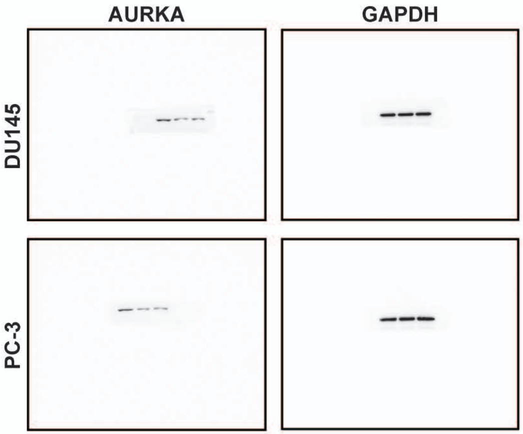

Fig. 5F

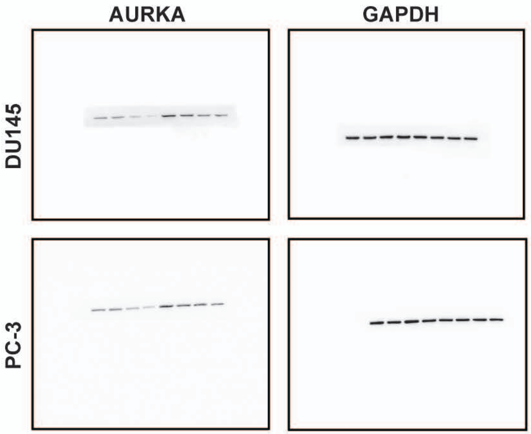

Fig. 5B

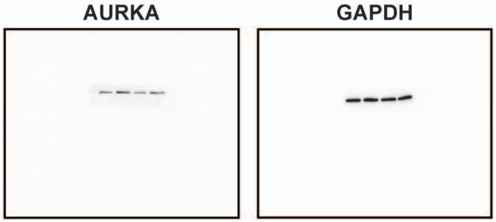

Fig. 5G

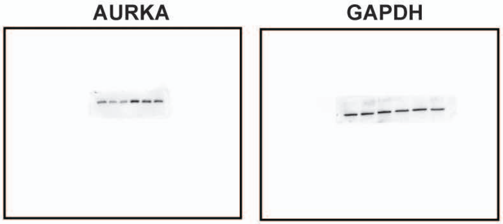

Fig. 5H

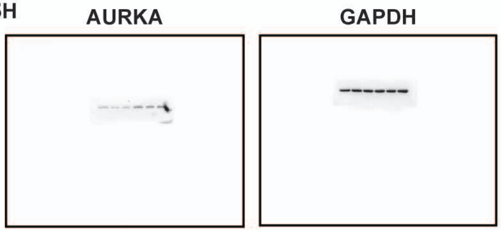

Fig. 4I

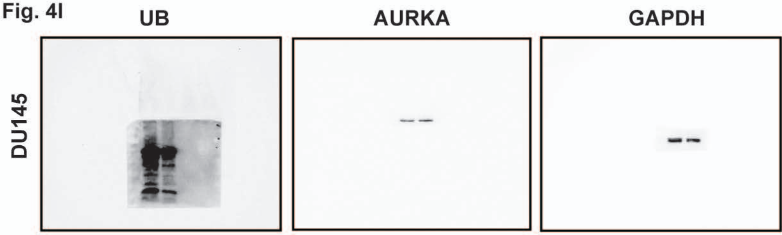

Fig. 4I

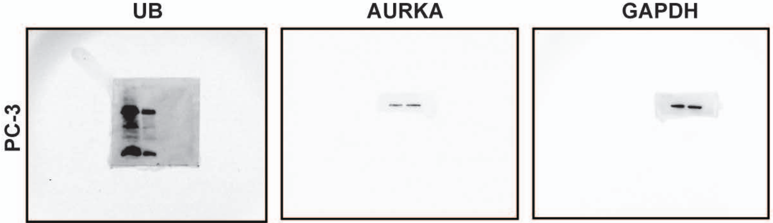

Fig. 6E

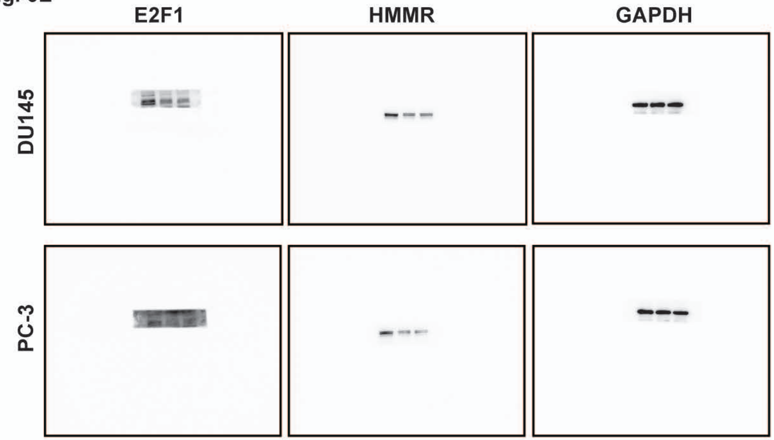

Fig. 6I

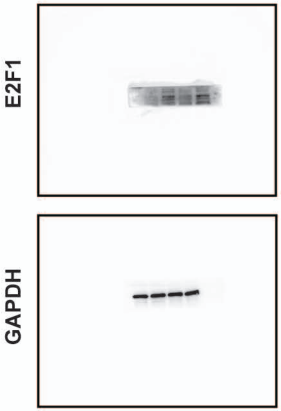

Supplementary Fig. S2A

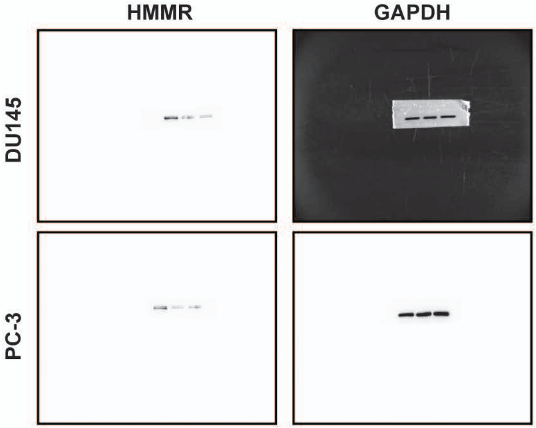

Supplementary Fig. S3

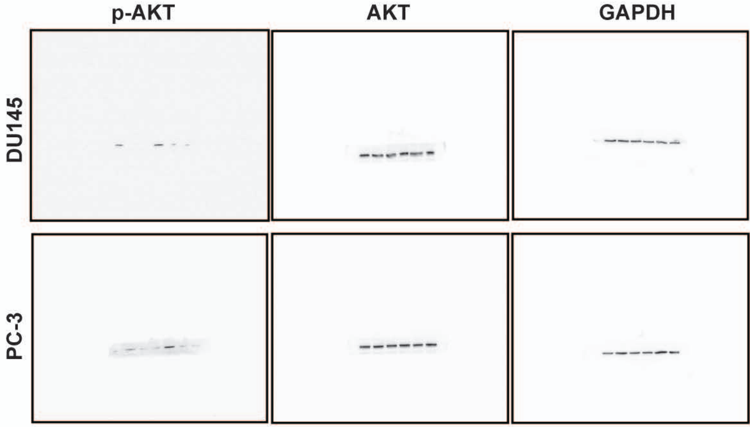

Supplementary Fig. S4D

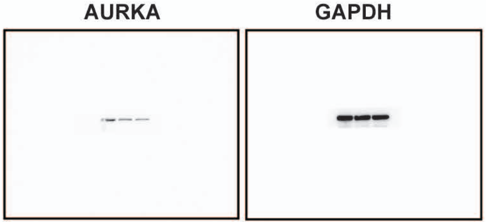

Supplementary Fig. S4E

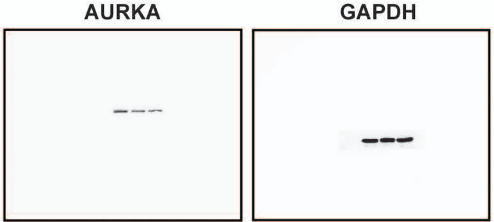

Supplement: Supplementary file 1 — Original western blotting [file 41420_2023_1341_MOESM1_ESM.pdf]
