# Supplementary material for: HMMR promotes prostate cancer proliferation and metastasis via AURKA/mTORC2/E2F1 positive feedback loop
Source: Cell Death Discov. 2023 Feb 7;9:48. doi: 10.1038/s41420-023-01341-0 (PMC9905489; doi:10.1038/s41420-023-01341-0)
Supplement: Supplementary file 2 — Supplementary materials and methods [file 41420_2023_1341_MOESM2_ESM.docx]

**Supplementary materials and methods**

**RNAi transfection, mRNA extraction and qRT–PCR**

Lipofectamine iMAX (Invitrogen, USA) was used for cell transfection following the protocol. For mRNA extraction, prepared cells were collected at the indicated times and lysed with lysis buffer provided by an RNA-Quick Purification Kit (YiShan Biotech, Shanghai, China). Total mRNA was isolated according to the protocol. After preparing the mRNA wells, cDNA synthesis was performed using the PrimeScript RT Reagent Kit (Takara, Shiga, Japan), and mRNA expression for specific targets was detected using TB Green Premix Ex Taq II (Takara, Shiga, Japan).

**Cell Counting Kit-8 (CCK-8)**

PCa cells after corresponding treatment digested, counted and resuspended. 1000 cells per well seeded into 96-well plate for culture until adhered. Cell Counting Kit-8 (APExBIO, USA) stock solution (10 ul) was added to each well and cultured for 2 hours, and then the absorbance value at 450nm for each well was measured through Spark 10M (Tecan, Austria). Following this process to continuously examine for 5 days and the growth index was calculated.

**Colony formation**

The treated cells were prepared as described above. 1000 cells were seeded into 6-well plate for culture. After cultured for 2-3 weeks, replaced medium with paraformaldehyde (Servicebio, Wuhan, China) for incubation at room temperature for 30 min. Staining cells with 0.1% crystal violet at room temperature for 15 min and washed twice with PBS. After drying naturally, clones were photographed and analyzed with AID vSpot Spectrum (AID, Germany).

**Flow cytometry (FCM) and cell cycle analysis**

The treated cells were prepared as described above. Cells were collected and washed with ice-cold PBS for three times and fixed with 80% iced ethanol at -20℃ for overnight. Removed ethanol and washed cells with ice-cold PBS for three times and treated cells with propidium iodide (PI) and RNase provided by Cell Cycle and Apoptosis Analysis Kit (YEASEN, China) for 30 min in the dark. The ratio of cell cycle phase was measured by the CytExpert (Beckman coulter, USA).

**Transwell assay**

The treated cells were prepared as described above. Chambers (8 μm pore size, Falcon, USA) and Matrigel (BD Science, USA) were used. For migration and invasion, 5 × 10^4 cells were resuspended with 200 ul serum-free medium and added into upper chambers (without matrigel for migration and with matrigel for invasion), with 700 ul medium supplemented 10% serum in the lower chambers, and cultured for indicated time. Fixed cells with paraformaldehyde (Servicebio, Wuhan, China) for 30 min after washed with PBS for twice. And stained cells with 0.1% crystal violet at room temperature for 10 min and removed cell which in upper chambers by swab. The images were captured with Leica DM2000 microscope (Leica Camera AG, Wetzlar, Germany).

**Wound healing**

Treated cells as required in 6-well plate and cultured until spread all over the plate. The 200 ul pipette was used to line the plate vertically to generate wound, and then wash cells softly with PBS. The images at initial time were captured with Olympus IX2 inverted microscope (Olympus, Tokyo, Japan). Cultured cells with serum-free medium for suggested time and images were captured at the end of time with Olympus IX2 inverted microscope (Olympus, Tokyo, Japan). The distance of cell moving and wound closure were analyzed.

**Dual-luciferase reports assay**

HEK-293T cells (8 × 10^4) were used for the detection of fluorescence. Luciferase reporter plasmids (psi-CHECK2) containing AURKA promotor region were constructed and cotransfected with HMMR overexpressing vector. Luciferase activity was measured using a dual luciferase assay kit (Promega, WI, USA) according to the protocol. Renilla and firefly luciferase (Rluc) intensity was measured using a SPARK 10 M spectrophotometer (Tecan, Austria).

**Antibodies regents for Western Blotting**

Antibodies used in this study were listed as follows: anti-HMMR (87129S, 1:1000), anti-mTOR (2983S, 1:1000), anti-phospho-mTOR (Ser2448, 5536S, 1:1000), anti-Akt (4691S, 1:1000), anti-phospho-Akt (Ser473, 4060S, 1:1000), anti-p21 (2947S, 1:1000), anti-CDK4 (12790S, 1:1000), anti-CDK6 (13331S, 1:1000), anti-Cyclin D1 (55506S, 1:1000), anti-AURKA (14475S, 1:1000), anti-E2F1 (3742S, 1:1000), anti-rabbit (7074, 1:5000) and anti-mouse (7076S, 1:5000) IgG HRP-linked antibodies purchased from Cell Signaling Technology (MA, USA). Anti-ubiquitin polyclonal antibody (10201-2-AP, 1:1000), anti-snail (13099-1-AP, 1:1000), anti-vimentin (10366-1-AP, 1:1000), anti-E-cadherin (20874-1-AP, 1:1000), anti-N-cadherin (22018-1-AP, 1:1000), anti-Flag monoclonal antibody (66008-3-Ig, 1:1000), and anti-His-Tag monoclonal antibody (66005-1-Ig, 1:1000) were purchased from Proteintech (Wuhan, China). Anti-GAPDH antibody (ab181602, 1:10 000) was purchased from Abcam (Cambridge, UK).
