# Supplementary material for: HMMR promotes prostate cancer proliferation and metastasis via AURKA/mTORC2/E2F1 positive feedback loop
Source: Cell Death Discov. 2023 Feb 7;9:48. doi: 10.1038/s41420-023-01341-0 (PMC9905489; doi:10.1038/s41420-023-01341-0)
Supplement: Supplementary file 3 — Supplementary table 1 [file 41420_2023_1341_MOESM3_ESM.docx]

**Supplementary table 1. The oligonucleotides transfected in this study are listed as follows.**

| Oligonucleotides | Sequence (5’-3’) |
| --- | --- |
| si-Con sense | UUCUCCGAACGUGUCACGUTT |
| si-Con antisense | ACGUGACACGUUCGGAGAATT |
| si-*HMMR*#1 sense | GACCAGGACUAAUGAACUATT |
| si-*HMMR*#1 antisense | UAGUUCAUUAGUCCUGGUCTT |
| si-*HMMR*#2 sense | CACUGGAUGAGCUUGAUAATT |
| si-*HMMR*#2 antisense | UUAUCAAGCUCAUCCAGUGTT |
| sh-*HMMR*#1 sense | GACCAGGACUAAUGAACUATT |
| sh-*HMMR*#1 antisense | UAGUUCAUUAGUCCUGGUCTT |
| si-Con sense | UUCUCCGAACGUGUCACGUTT |
| si-Con antisense | ACGUGACACGUUCGGAGAATT |
| si-*AURKA*#1 sense | CCAGCGCAUUCCUUUGCAATT |
| si- *AURKA*#1 antisense | UUGCAAAGGAAUGCGCUGGTT |
| si- *AURKA*#2 sense | GCAGAGAACUGCUACUUAUTT |
| si- *AURKA*#2 antisense | AUAAGUAGCAGUUCUCUGCTT |
| si-Con sense | UUCUCCGAACGUGUCACGUTT |
| si-Con antisense | ACGUGACACGUUCGGAGAATT |
| si-*E2F1*#1 sense | CCUGGAAACUGACCAUCAGTT |
| si-*E2F1*#1 antisense | CUGAUGGUCAGUUUCCAGGTT |
| si-*E2F1*#2 sense | GGACUCUUCGGAGAACUUUTT |
| si-*E2F1*#2 antisense | AAAGUUCUCCGAAGAGUCCTT |
