# Supplementary material for: HMMR promotes prostate cancer proliferation and metastasis via AURKA/mTORC2/E2F1 positive feedback loop
Source: Cell Death Discov. 2023 Feb 7;9:48. doi: 10.1038/s41420-023-01341-0 (PMC9905489; doi:10.1038/s41420-023-01341-0)
Supplement: Supplementary file 4 — Supplementary table 2 [file 41420_2023_1341_MOESM4_ESM.docx]

**Supplementary table 2. The primers used in this study are listed as follows.**

| Primers | Sequence (5’-3’) |
| --- | --- |
| *HMMR* Forward | AGAACCAACTCAAGCAACAGG |
| *HMMR* Reverse | AGGAGACGCCACTTGTTAATTTC |
| *P21* Forward | AGGTGGACCTGGAGACTCTCAG |
| *P21* Reverse | TCCTCTTGGAGAAGATCAGCCG |
| *CDK4* Forward | CCATCAGCACAGTTCGTGAGGT |
| *CDK4* Reverse | TCAGTTCGGGATGTGGCACAGA |
| *CDK6* Forward | GGATAAAGTTCCAGAGCCTGGAG |
| *CDK6* Reverse | GCGATGCACTACTCGGTGTGAA |
| *E-cadherin* Forward | GCCTCCTGAAAAGAGAGTGGAAG |
| *E-cadherin* Reverse | TGGCAGTGTCTCTCCAAATCCG |
| *N-cadherin* Forward | CCTCCAGAGTTTACTGCCATGAC |
| *N-cadherin* Reverse | GTAGGATCTCCGCCACTGATTC |
| *Snail* Forward | TGCCCTCAAGATGCACATCCGA |
| *Snail* Reverse | GGGACAGGAGAAGGGCTTCTC |
| *Vimentin* Forward | AGGCAAAGCAGGAGTCCACTGA |
| *Vimentin* Reverse | ATCTGGCGTTCCAGGGACTCAT |
| *AURKA* Forward | GAGGTCCAAAACGTGTTCTCG |
| *AURKA* Reverse | ACAGGATGAGGTACACTGGTTG |
| *E2F1* Forward | GGACCTGGAAACTGACCATCAG |
| *E2F1* Reverse | CAGTGAGGTCTCATAGCGTGAC |
| *GAPDH* Forward | GTCTCCTCTGACTTCAACAGCG |
| *GAPDH* Reverse | ACCACCCTGTTGCTGTAGCCAA |
| *HMMR promotor* Forward | AAACTGAACACAAGCCCCGA |
| *HMMR promotor* Reverse | CATTGAAGTTCAGGTGCCGC |
