# Supplementary figures and images for: HMMR promotes prostate cancer proliferation and metastasis via AURKA/mTORC2/E2F1 positive feedback loop
Source: Cell Death Discov. 2023 Feb 7;9:48. doi: 10.1038/s41420-023-01341-0 (PMC9905489; doi:10.1038/s41420-023-01341-0)

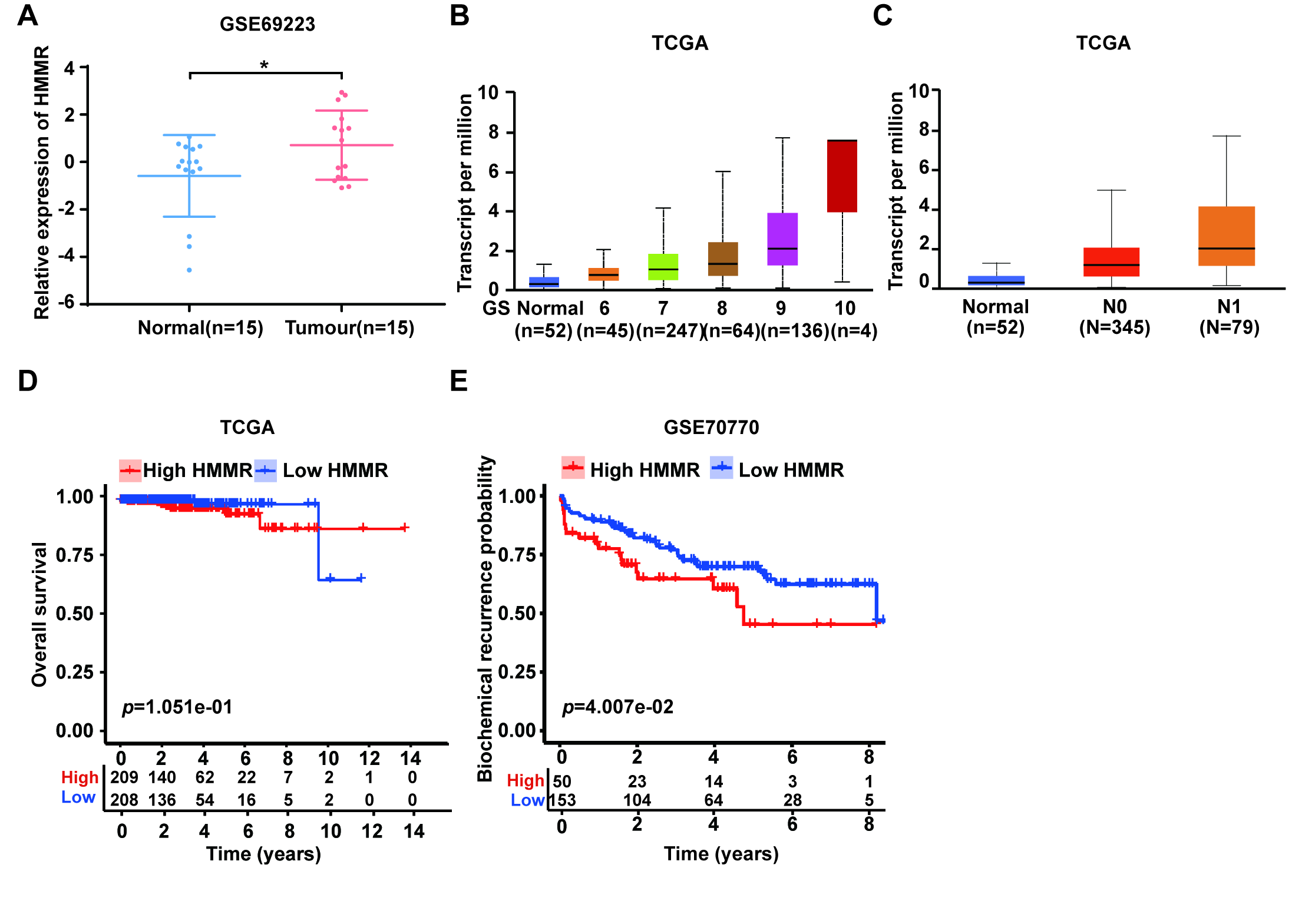

Supplement: Supplementary file 5 — Supplementary Fig. S1 [file 41420_2023_1341_MOESM5_ESM.tif]

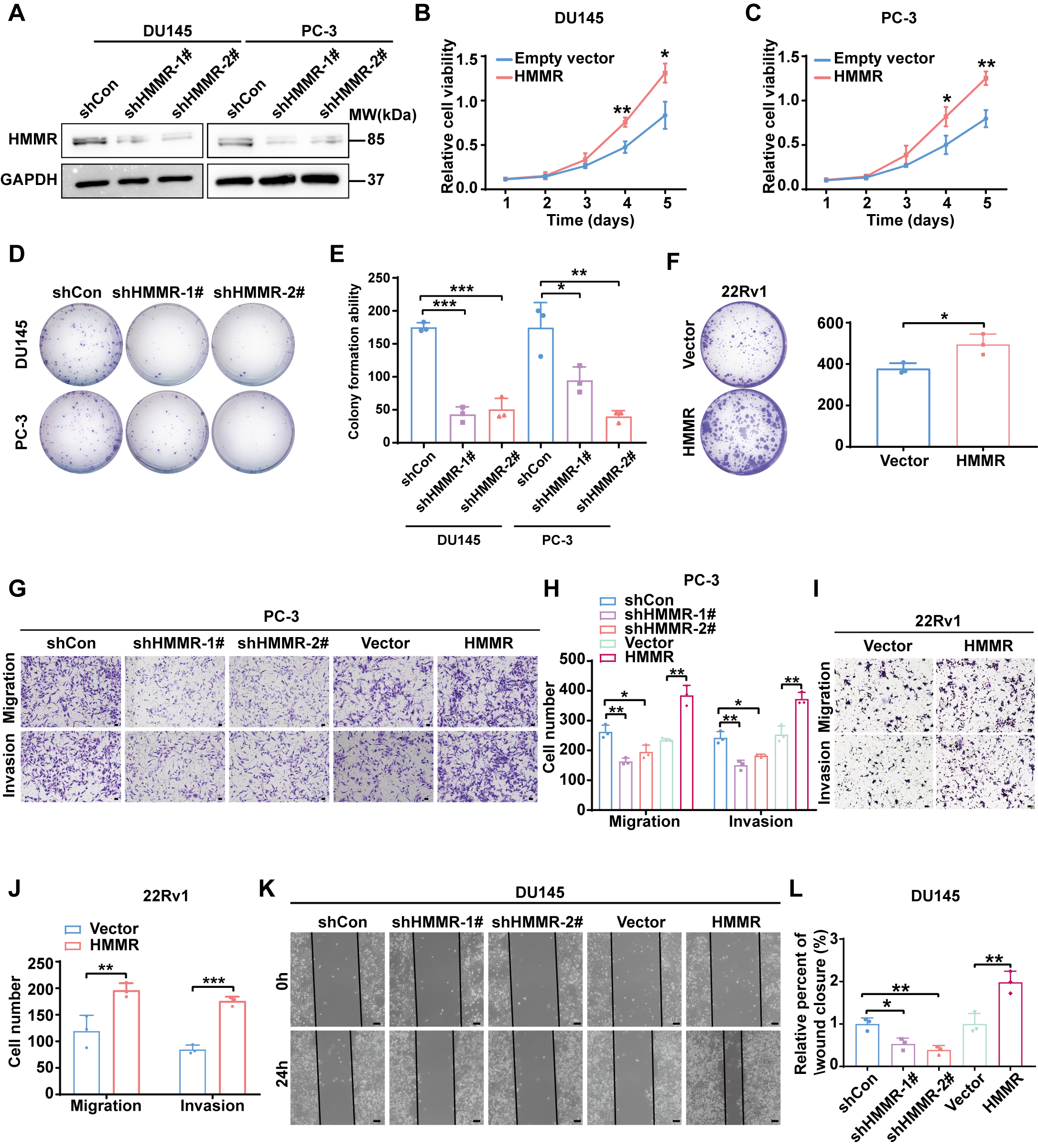

Supplement: Supplementary file 6 — Supplementary Fig. S2 [file 41420_2023_1341_MOESM6_ESM.tif]

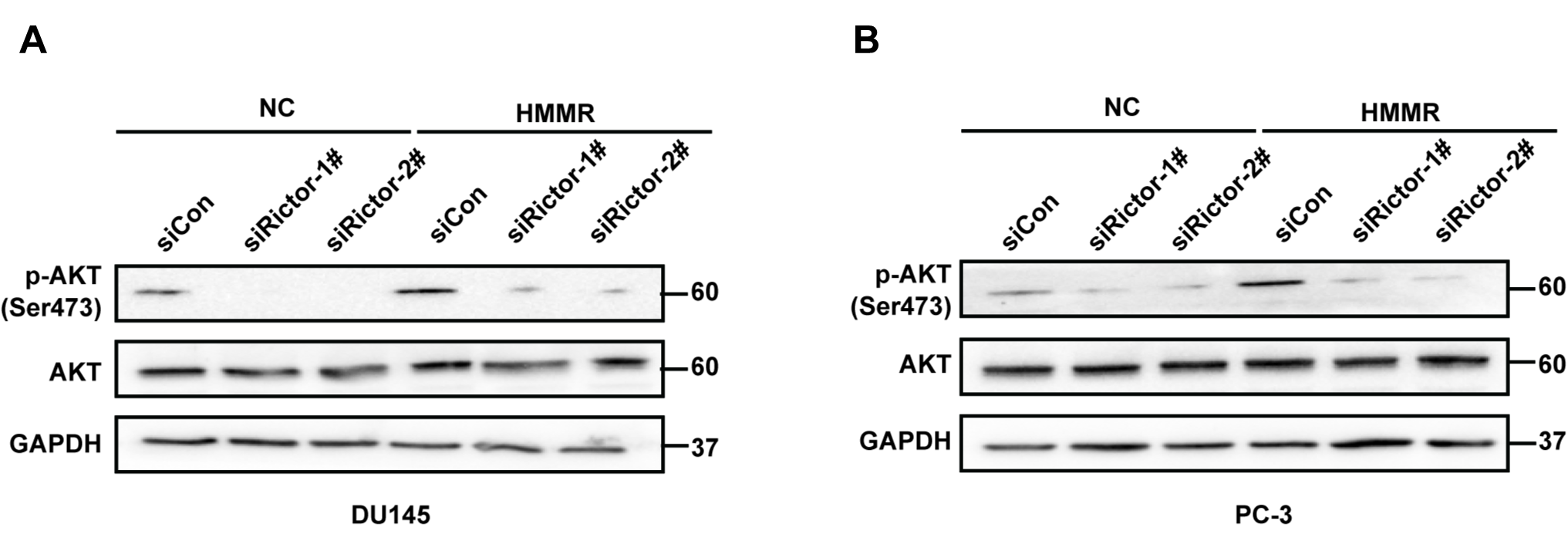

Supplement: Supplementary file 7 — Supplementary Fig. S3 [file 41420_2023_1341_MOESM7_ESM.tif]

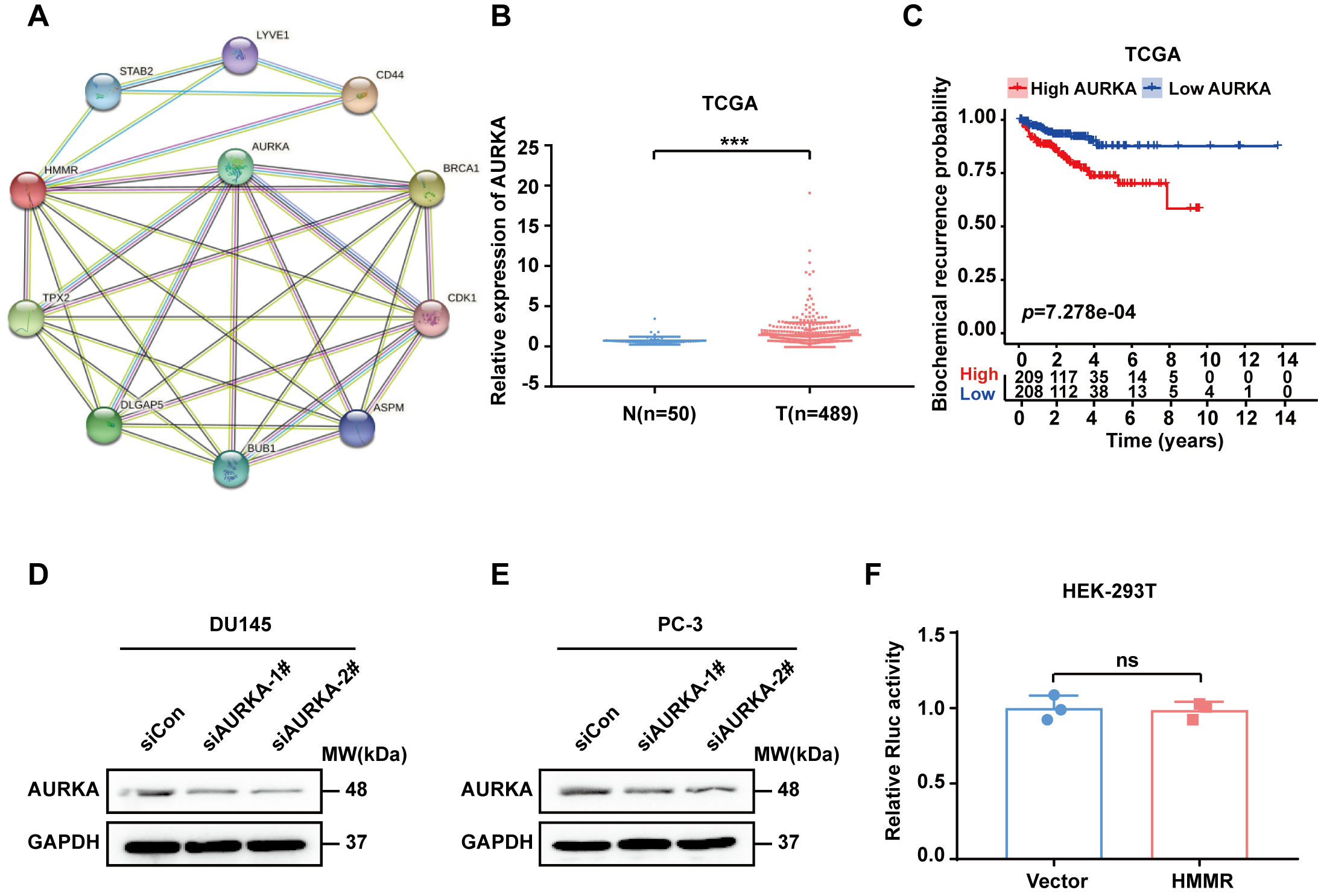

Supplement: Supplementary file 8 — Supplementary Fig. S4 [file 41420_2023_1341_MOESM8_ESM.tif]

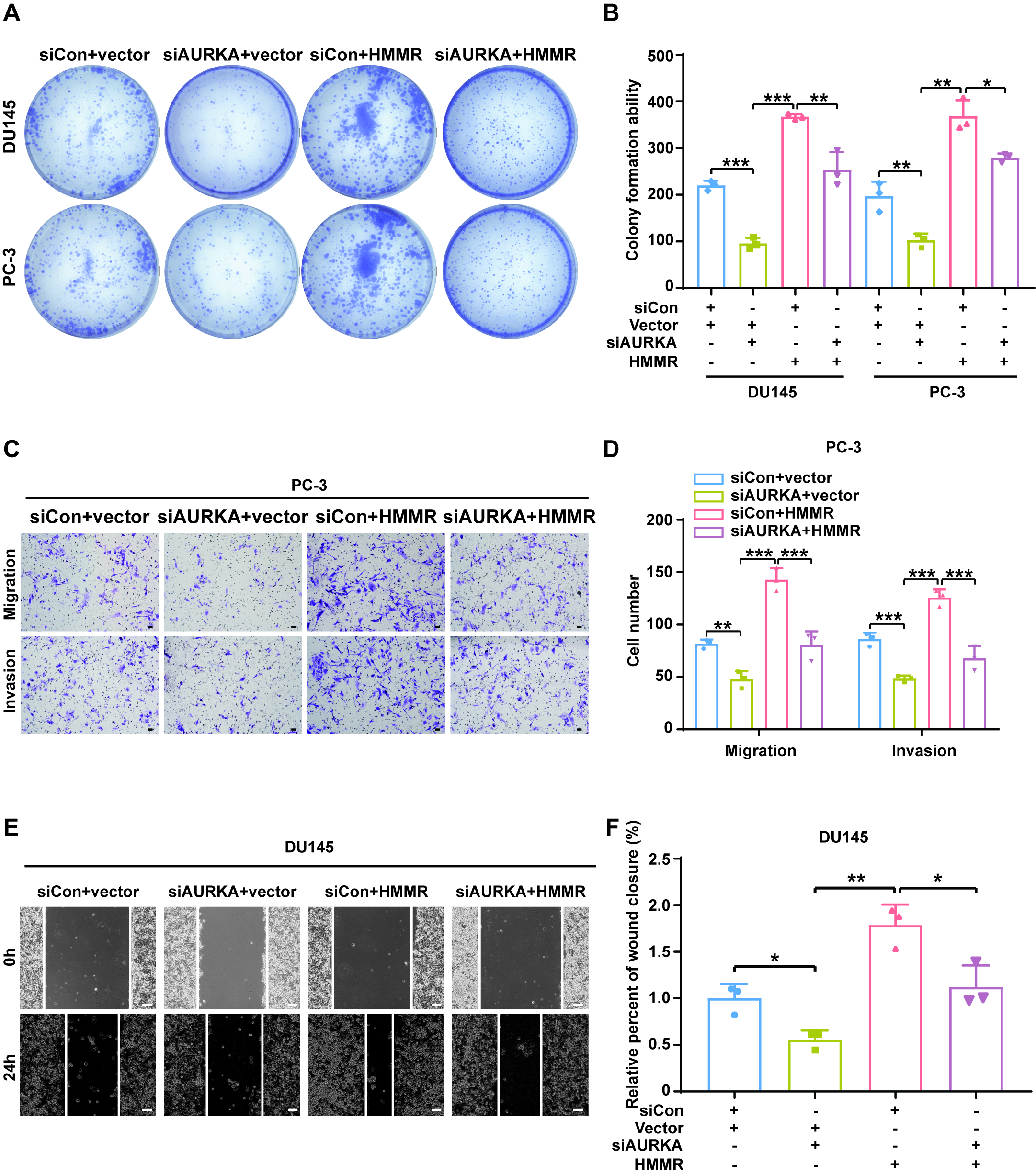

Supplement: Supplementary file 9 — Supplementary Fig. S5 [file 41420_2023_1341_MOESM9_ESM.tif]

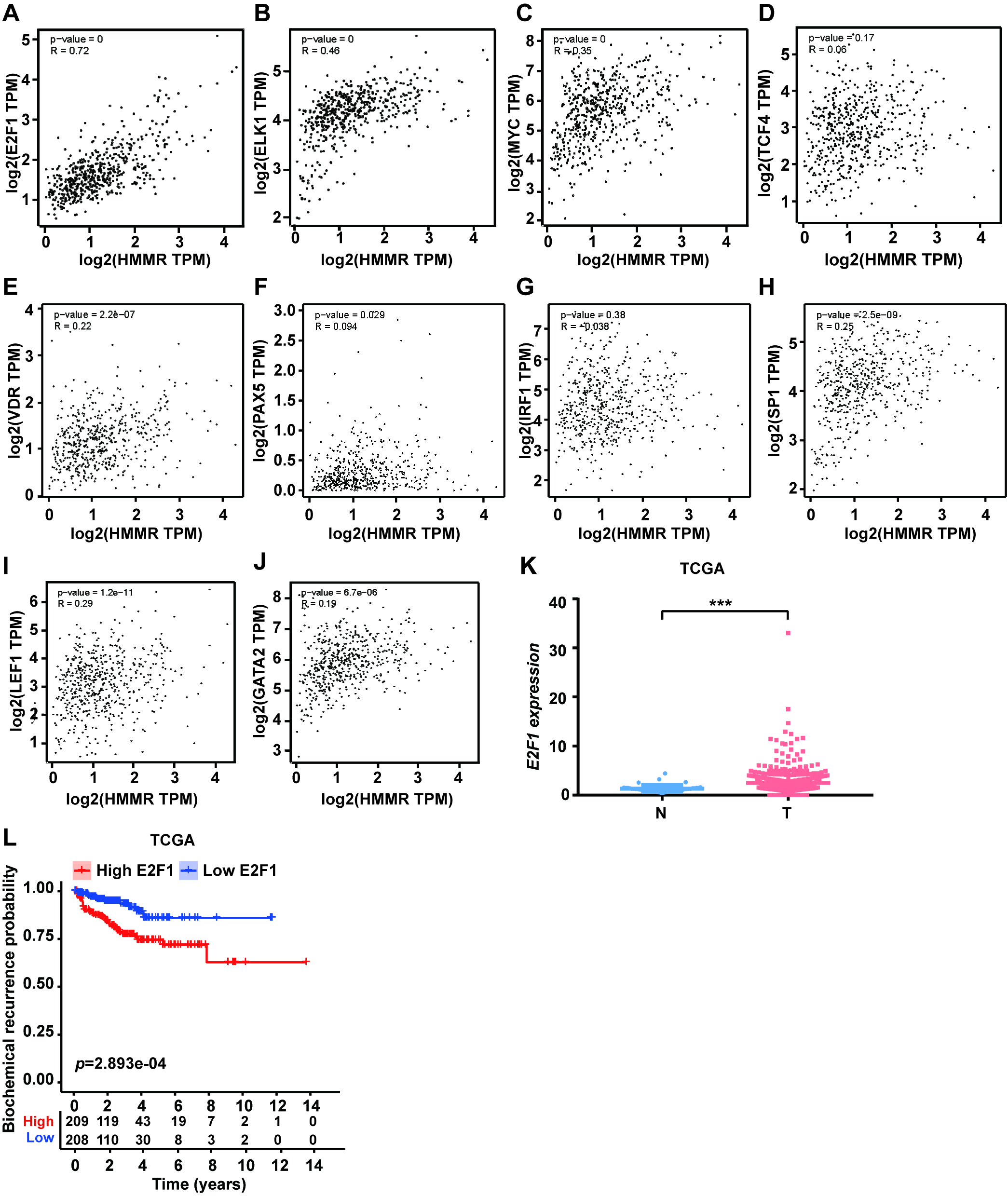

Supplement: Supplementary file 10 — Supplementary Fig. S6 [file 41420_2023_1341_MOESM10_ESM.tif]

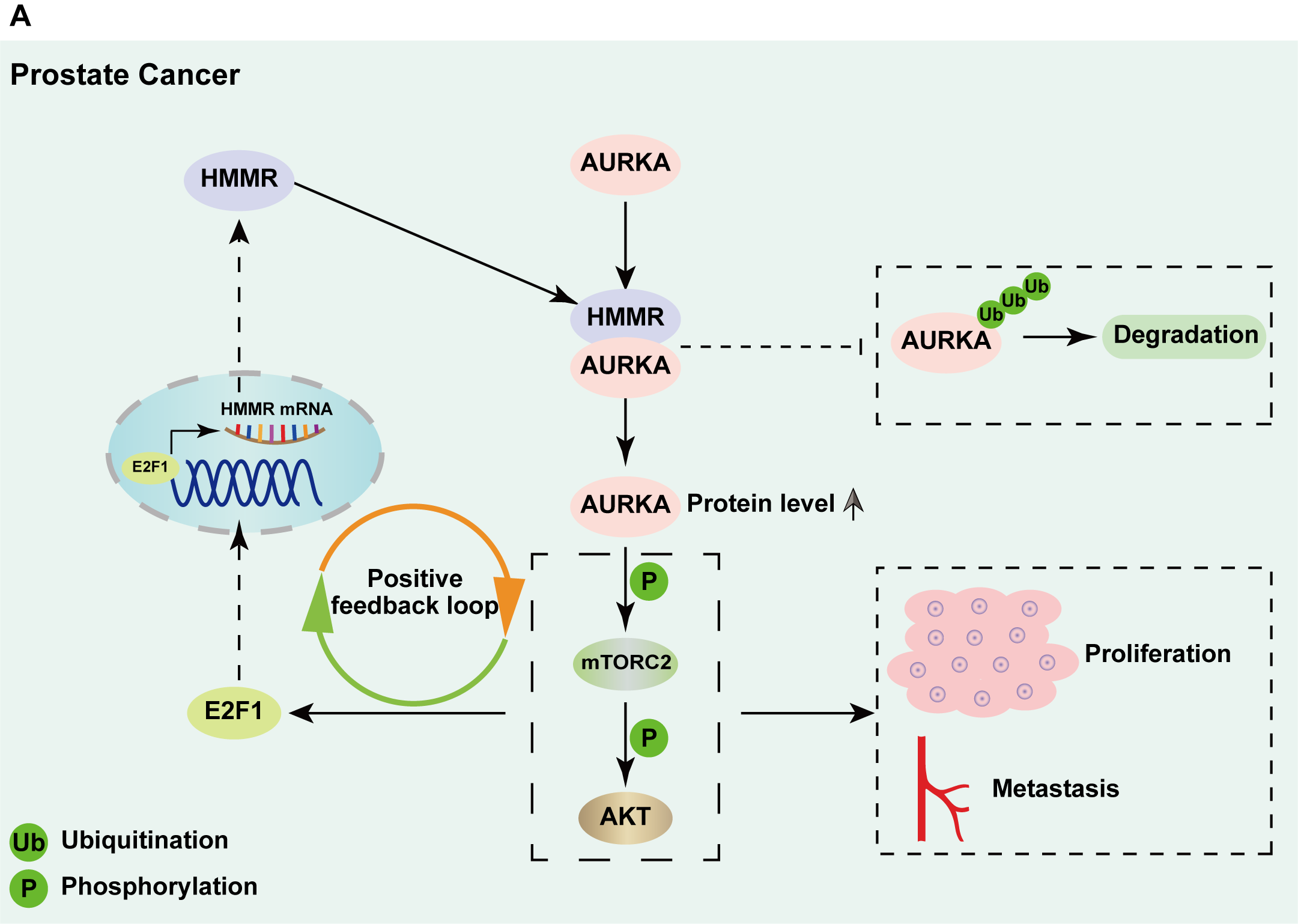

Supplement: Supplementary file 11 — Supplementary Fig. S7 [file 41420_2023_1341_MOESM11_ESM.tif]
