# Supplementary material for: HMMR promotes prostate cancer proliferation and metastasis via AURKA/mTORC2/E2F1 positive feedback loop
Source: Cell Death Discov. 2023 Feb 7;9:48. doi: 10.1038/s41420-023-01341-0 (PMC9905489; doi:10.1038/s41420-023-01341-0)
Supplement: Supplementary file 12 — Supplementary figure legends [file 41420_2023_1341_MOESM12_ESM.docx]

**Supplementary figure legends**

**Fig. S1** **HMMR was upregulated in PCa and associated with poor prognosis**. **A** Expression of HMMR in PCa and normal tissues (GSE69233). **B-C** Expression of HMMR in differential stage PCa tissues based on Gleason score (**B**) and lymph node tissue (**C**). **D** Overall survival (OS) of patients with PCa based on HMMR expression (High and low group). **E** Biochemical recurrence of PCa based on HMMR expression in GEO dataset (GSE70770). Data were presented as mean±SD. **p*<0.05.

**Fig. S2** **HMMR promoted PCa progression in vitro and in vivo. A** The HMMR knockdown efficiency were verified through Western blot. **B-C** CCK-8 analysis of cell viability after transfecting DU145 (**B**) and PC-3 (**C**) cells with HMMR-overexpressing plasmid or vector at the indicated times. **D** Analysis of colony formation in DU145 and PC-3 cells after transfecting HMMR specific siRNA. **E** Statistics of proliferation index of colony formation. **F** Analysis of colony formation in 22Rv1 cells after upregulating HMMR. **G-H** Representative images of the Transwell assay for evaluating the migration and invasion of PC-3 cells with stable HMMR silencing (shHMMR-1#, shHMMR-2#) or overexpression (HMMR plasmid). shCon and vector were used as negative controls, scale bar: 100 μm. **I-J** Representative images of the Transwell assay for evaluating the migration and invasion of 22Rv1 cells with HMMR upregulation. Vector were used as negative controls, scale bar: 100 μm. **K** Analysis of wound healing of DU145 cells after transfecting HMMR specific siRNA or overexpression vector. **L** Statistics of wound closure rate in HMMR silencing or overexpression DU145 cells. Data were presented as mean±SD. **p*<0.05, ***p*<0.01, ****p*<0.001.

**Fig. S3** **HMMR induced AKT activation was in mTORC2 dependent manner.** **A- B** Western blotting detected the level of p-AKT(Ser473) after Rictor knock down in control group or HMMR overexpression group.

**Fig. S4 Association between HMMR and AURKA. A** Interaction of HMMR with potential partners, the results were analyzed by STRING. **B** Expression of AURKA in PCa and normal tissues (TCGA). **C** Prognosis of PCa patients with BCR: patients were stratified by HMMR expression. **D-E** Analysis of AURKA silencing efficiency in DU145 and PC-3 cells by transfecting AURKA specific siRNA. **F** Luciferase activity were used to analyse the interaction between HMMR and AURKA promoter region. Data were presented as mean±SD. ****p*<0.001, ns: not significant.

**Fig. S5 Oncogenic role of HMMR partially dependent on AURKA. A-B** Analysis of colony formation of four groups in DU145 and PC-3 cells after transfecting AURKA specific siRNA and/or HMMR overexpression vector (siCon+vector, siAURKA+vector, siCon+HMMR, siAURKA+HMMR). **C-D** Analysis of transwell of PC-3 cells in four groups (siCon+vector, siAURKA+vector, siCon+HMMR, siAURKA+HMMR). **E-F** Analysis of wound healing of DU145 cells in four groups (siCon+vector, siAURKA+vector, siCon+HMMR, siAURKA+HMMR). The siCon and vector were used as negative control respectively. Data were presented as mean±SD. **p*<0.05, ***p*<0.01, ****p*<0.001.

**Fig. S6 Association between HMMR and potential transcription factors. A-J** Correlation of HMMR and candidate transcription factors in TCGA through GEPIA analysis. **K** Expression of E2F1 in TCGA, tumour tissues versus normal tissues. **L** Survival of PCa patients (BCR) in E2F1 high and low groups in TCGA. Data were presented as mean±SD. ****p*<0.001.

**Fig. S7 Schematic diagram of the HMMR mediated positive feedback loop consisting of AURKA/mTORC2/E2F1 in PCa progression**. **A** Schematic diagram of the mechanism underlying the positive feedback loop between HMMR and E2F1 mediated by AURKA/mTORC2/AKT pathway in regulating PCa proliferation, migration and invasion.
